# The Role of Coral-Associated Bacterial Communities in Australian Subtropical White Syndrome of *Turbinaria mesenterina*

**DOI:** 10.1371/journal.pone.0044243

**Published:** 2012-09-06

**Authors:** Scott Godwin, Elizabeth Bent, James Borneman, Lily Pereg

**Affiliations:** 1 Research Centre for Molecular Biology, School of Science and Technology, University of New England, Armidale, NSW, Australia; 2 Department of Plant Pathology and Microbiology, University of California Riverside, Riverside, California, United States of America; 3 Biodiversity Institute of Ontario, Department of Integrative Biology, University of Guelph, Guelph, Ontario, Canada; University of California Merced, United States of America

## Abstract

Australian Subtropical White Syndrome (ASWS) is an infectious, temperature dependent disease of the subtropical coral *Turbinaria mesenterina* involving a hitherto unknown transmissible causative agent. This report describes significant changes in the coral associated bacterial community as the disease progresses from the apparently healthy tissue of ASWS affected coral colonies, to areas of the colony affected by ASWS lesions, to the dead coral skeleton exposed by ASWS. In an effort to better understand the potential roles of bacteria in the formation of disease lesions, the effect of antibacterials on the rate of lesion progression was tested, and both culture based and culture independent techniques were used to investigate the bacterial communities associated with colonies of *T. mesenterina*. Culture-independent analysis was performed using the Oligonucleotide Fingerprinting of Ribosomal Genes (OFRG) technique, which allowed a library of 8094 cloned bacterial 16S ribosomal genes to be analysed. Interestingly, the bacterial communities associated with both healthy and disease affected corals were very diverse and ASWS associated communities were not characterized by a single dominant organism. Treatment with antibacterials had a significant effect on the rate of progress of disease lesions (p = 0.006), suggesting that bacteria may play direct roles as the causative agents of ASWS. A number of potential aetiological agents of ASWS were identified in both the culture-based and culture-independent studies. In the culture-independent study an Alphaproteobacterium closely related to *Roseovarius crassostreae*, the apparent aetiological agent of juvenile oyster disease, was found to be significantly associated with disease lesions. In the culture-based study *Vibrio harveyi* was consistently associated with ASWS affected coral colonies and was not isolated from any healthy colonies. The differing results of the culture based and culture-independent studies highlight the importance of using both approaches in the investigation of microbial communities.

## Introduction

The incidence of diseases of scleractinian (reef building) corals and other reef organisms has increased over the past 20 years, resulting in drastic changes to some reef ecosystems, including total loss of coral cover in some locations [1], [2]. As many as 30 distinct diseases of scleractinian corals have been reported worldwide [3], [4], [5], with microbial organisms implicated as the causative agent in almost half of these reported diseases. As a result, increased efforts have been made to understand the diversity, structure and function of coral associated microbial communities [6].

*Turbinaria mesenterina* is an ecologically important scleractinian coral that is abundant on shallow rocky reefs in subtropical eastern Australia, where it comprises a large proportion of the benthic cover [7], [8]. In 2000, a newly observed syndrome was reported affecting *T*. *mesenterina* and other coral species in the Solitary Islands Marine Park (SIMP), NSW, Australia. This syndrome presents as a typical ‘white syndrome’ with gradual loss of living tissue which may begin at any point on the coral colony as a small patch of missing tissue, before spreading outwards, forming a lesion of exposed white skeleton [9]. Preliminary microscopic investigations of these lesions revealed large numbers of motile bacteria at the interface between apparently healthy tissue and exposed skeleton [10]. Later, Dalton *et al*. [10] used field and aquarium experiments to demonstrate that the syndrome is a temperature-dependant, infectious disease, which can be transmitted by direct contact between coral colonies, and possibly via a predatory vector [11].

The fact that this disease is clearly infectious and transmissible between coral colonies, is associated with the presence of a bacterial community and has a distinct geographical and host range distinguishes it from phenotypically similar, but apparently non-infectious, diseases of other coral taxa observed on the Great Barrier Reef [4]. The disease was therefore named Australian Subtropical White Syndrome (ASWS) [10] to differentiate it from other coral diseases which may appear superficially similar.

Although it was clear that ASWS is infectious, the identity of the causative agent involved was still unknown. At the present time most of the diseases of scleractinian corals that cause progressively expanding lesions of tissue lysis with phenotypic characteristics similar to ASWS appear to involve bacterial pathogens [12], [13], [14], [15], [16], [17], [18], [19], [20], [21]. Although the possibility of a viral pathogen has not been ruled out, in preliminary investigations it has been considered possible that ASWS also has a bacterial aetiology, based on the microscopic observation of large numbers of motile bacterial cells in association with diseased tissue, the fact that it is transmissible between colonies and the fact that no clear evidence of proliferation of fungal or protistan pathogens has been observed [10].

The aim of this study was to investigate the role of bacteria in the development and progression of ASWS in *T. mesenterina* and to identify potential causative agents. The first specific objective was to compare and contrast the bacterial communities associated with healthy and ASWS affected *T. mesenterina* colonies in order to identify potential causative agents of the disease. The structure of the bacterial communities were investigated by constructing comprehensive profiles of the bacterial communities associated with both healthy and ASWS affected *T. mesenterina* using a 16S ribosomal gene based culture independent approach. The oligonucleotide fingerprinting of ribosomal genes method (OFRG) [22] was employed for the culture-independent analysis allowing a large library of 16S rRNA gene clones to be comprehensively analysed without performing nucleotide sequencing on the entire library. The second specific objective of this study was to isolate ASWS associated bacterial strains in culture so that their potential roles as pathogens could be assessed. Non-selective media and conditions were chosen to allow a wide range of isolates to be obtained. The last objective was to experimentally test the hypothesis that ASWS lesions are caused by infection with a bacterial pathogen. This was done by testing the effect of antibacterial compounds on the rate of progression of lesions.

Comparison of the bacterial community profiles obtained from healthy versus ASWS affected coral colonies identified a number of bacteria potentially implicated as disease causing agents and will assist future efforts to definitively identify pathogens through manipulative experiments with live coral specimens and bacterial cultures. It is hoped that identification of the causative agents of ASWS may lead to the formulation of management strategies for the disease which could be integrated into a reef management plan.

## Materials and Methods

### Sample Collection

**Figure 1:**
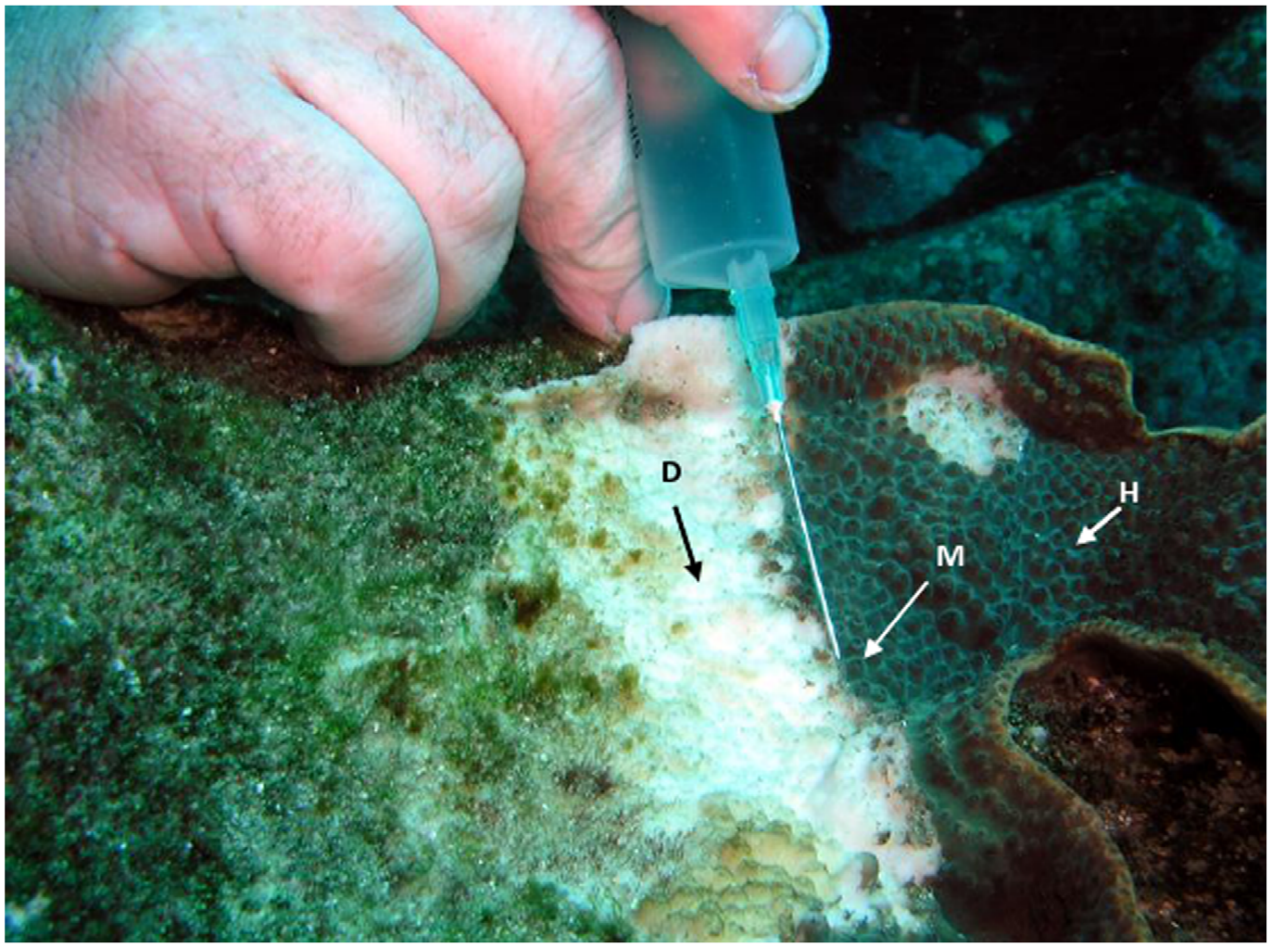
*T. mesenterina* colonies displaying typical signs of ASWS. Arrows indicate the regions sampled for bacterial community analysis. Dark areas of the coral surface are covered in living tissue, white areas are recently exposed calcium carbonate skeleton. (H) – Apparently healthy tissue of disease colony, (M) – Margin of disease lesion, (D) – Dead coral skeleton. Healthy tissue from a nearby colony unaffected by disease (C) was also collected (not visible in this photograph).

Samples for this study were collected under a NSW DPI Scientific Research Permit (permit number P06/0064). All samples for bacterial community analyses were collected in autumn between the months of March and May in 2004 and 2005 from rocky reefs at depths of 10–20 m by SCUBA diving. Diseased coral colonies were identified by visual inspection. When selecting diseased coral samples, only colonies that displayed an actively advancing disease lesion with a clean area of recently exposed white aragonite skeleton were selected for sampling (Figure 1). Older lesions that were no longer advancing were identified by the presence of algae colonization on the exposed coral skeleton, or by the presence of a lighter coloured margin of new coral tissue growth. Healthy coral colonies were defined as those with no visible signs of disease lesions, bleaching (loss of colour), predation scars or sediment damage. Samples for the culture-based and culture-independent studies were collected separately from different colonies. For each study, a total of 3 ASWS affected and 2 apparently healthy colonies were sampled. Samples were collected from the active disease margin of ASWS affected *T. mesenterina* colonies, and from the apparently healthy and dead regions 30–40 mm on either side of the margin (Figure 1). The apparently healthy tissues of ASWS affected colonies were inspected to verify that they included living polyps and that tissue was not sloughing off the skeleton. Samples of the dead coral skeleton were taken from areas with no visible signs of algal colonization. Samples were also collected from apparently healthy colonies with no signs of damage or disease located within a 5 m radius of the diseased colony.

For the culture-based study, samples were taken of both the coral mucus and tissues. Mucus samples were taken first by drawing a syringe with a 21 gauge needle attached along the surface of the coral (Figure 1). This was done to prevent loss of mucus and associated bacteria during subsequent collection of tissue samples. Tissue samples were taken by removing small sections (approximately 15 cm^2^) from the coral colony using a hammer and chisel. The syringes and coral fragments were placed immediately into separate clean Ziploc bags underwater. Upon surfacing, bags containing samples for isolation of culturable bacteria were placed in a 60 L container of seawater at the same ambient temperature as the water the samples were collected from. This was done to maintain a constant temperature during transport back to the laboratory and to minimize perturbation of the microbial community caused by rapid temperature changes or stress to the coral host.

Coral tissue samples for culture-independent molecular analysis were collected in the same way, but were transported on ice, rather than at ambient water temperature, and immediately transferred to −20°C upon return to the laboratory. Mucus samples were not collected for the culture-independent study. Coral samples were prepared for DNA extraction by freezing in liquid nitrogen and crushing into a fine powder in a mortar and pestle. The crushed coral was stored at −70°C. A total of 3 ASWS affected and 2 healthy colonies were sampled for the culture-independent study. The 11 samples in the final set consisted of three replicate samples of apparently healthy sections of diseased corals (**H**), two replicate samples of disease margins (**M**), four replicate samples of dead sections of diseased colonies (**D**) and two replicate samples from apparently healthy colonies (**C**) (Figure 1).

### Total Coral DNA Extraction and Amplification of Bacterial 16S rRNA Genes

DNA was extracted from coral samples using the Powersoil DNA isolation kit (MoBio, Carlsbad, CA). 250 mg of each ground coral sample was processed according to the manufacturer’s instructions. The resulting DNA preparation was used for PCR without any further processing.

16S rRNA genes were amplified from total coral DNA extracts in multiple 25 µL reactions containing 1X PCR buffer (Qiagen), 1 µL Q solution (a proprietary reagent designed to aid in the amplification of difficult samples (Qiagen), 25 mM MgCl_2_, 250 µM of each deoxynucleoside, 5U of *Taq* polymerase, 1 µL of coral DNA extract, and the forward primer (27F 5′**GGAGACAU**GAGCTCAGAGTTTGATCMTGGCTCAG) and reverse primer (1492R 5′**GGGAAAGU**CACGYTACCTTGTTACGACTT) at a concentration of 400 nM each. These primers included eight nucleotide modifications (indicated in bold) to allow cloning with the USER friendly cloning kit (New England Biolabs). PCR cycling parameters were 94°C for 2 minutes, followed by 35 cycles of 94°C for 30 s, 48°C for 30 s and 72°C for 2 min, with a final extension step of 72°C for 3 min. PCR products were quantified on agarose gels, and 4–5 replicate reactions were combined for each sample. The pooled product was evaporated to approximately 10 µL on a Savant Speed Vac (Savant). The 1.5 kb product corresponding to the amplified 16S rRNA gene was excised from 1.5% agarose gels and purified with a Qiagen MinElute kit (Qiagen).

### Clone Library Construction

Purified PCR products derived from the crushed coral samples were assembled into the pNEB205A vector (New England Biolabs) and transformed into *Escherichia coli* DH5α competent cells (Invitrogen, Carlsbad, California) by heat shock. Transformed cells were grown overnight at 37°C on Luria-Bertani (LB) agar containing 100 µg/mL ampicillin, 200 µg/mL isopropyl-β-D-1-thioglactopyranoside (IPTG) and 70 mg/mL 5-bromo-4-chloro-3-indolyl β-D-galactopyranoside (X-gal). For each of the 11 replicate coral samples (see Section 4.2.1 for sample categories), 768 white colonies were randomly selected using a QPix robotic colony picker (Genetix, Hampshire, UK) and transferred to 384 well culture plates containing 30 µL LB medium supplemented with 8% glycerol and 100 µg/mL ampicillin per well. Clones were grown in a shaking incubator (300 rpm) for 16 hours at 37°C. Clone libraries were stored at −70°C.

### PCR for Macroarray Construction and Printing

9600 clone arrays were constructed by spotting PCR amplified rRNA genes onto nylon membranes. To amplify the cloned genes, 1 µL portions of freshly grown overnight clone library cultures were transferred to 384 well PCR plates using a 384-pin split pin replicator (V&P Scientific, San Diego, California). Each well contained a 15 µL reaction mixture of 50 mM Tris (pH 8.3), 500 µg/mL bovine serum albumin, 2.5 mM MgCl_2_, 250 µM of each deoxynucleoside triphosphate 400 nm each of the forward primer (UserOFRGFor2–5′TCGAGCTCAGGCGCGCCTTATTAAGCTGA) and reverse primer (UserOFRGRev2–5′GCCAAGCTTCCTGCAGGGTTTAAACGCTGA) and 0.75U *Taq* polymerase. Plates were sealed with Thermo-seal foil (Marsh Bio Products) using a preheated Thermo-sealer (Abgene, Epsom, United Kingdom). PCR was performed by alternately submerging the sealed plates in waterbaths held at 72°C and 94°C. The cycling parameters were 94°C for 10 min followed by 35 cycles of 94°C for 1 min and 72°C for 2 min. A final extension step was performed at 72°C for 5 min. An additional library of 96 control clones with defined nucleotide sequences was required for analysis of the OFRG data. These clones were processed alongside the sample library in three replicate 384 well plates, each containing 4 replicates of the 96 control clones [22].

The PCR products were printed onto nylon membranes using a QPix robot (Genetix) as described by Bent et al. [22]. In addition to the 768 clones from each of the coral samples, 3 replicates of the 384 control clone library were included in the arrays, giving a total of 9600 clones per array. A total of 45 macroarrays (11 cm × 7 cm each) were printed. These replicate arrays were used in the hybridisation experiments.

### Array Hybridisation

38 separate hybridisation experiments were performed as described by Valinsky et al. [23]. The tenmer bacterial probes (Table 1) were designed using a previously described simulated annealing algorithm [24].

**Table 1:** Tenmer DNA probes used in hybridisation experiments.

Probe Name	Sequence	Probe Name	Sequence
Reference	GCTGCTGGCA	B64	GCTAACGCAT
B2	GGGCGAAAGC	B66	CATTCAGTTG
B5	GAGACAGGTG	B73	GTCTCAGTTC
B21	CCAGACTCCT	B74	AGGCTAGAGT
B25	CGTGGGGAGC	B79	CAACCCTTGT
B30	ACGTAATGGT	B80	GCGTGAGTGA
B31	TCCAGAGATG	B87	GGTGCAAGCG
B38	CCTTCGGGAG	B89	AGCTAGTTGG
B39	CCTACCAAGG	B92	AATACCGGAT
B42	GATGAACGCT	B94	TGTGGGAGGG
B44	GTGGGGTAAA	B98	CCCGCACAAG
B46	GGTAATGGCC	B99	CGGTACAGAG
B51	CCGTGAGGTG	B100	ACCAAGGCAA
B52	AGTCGAACGG	B101	TTGCCAGCGG
B56	TTGGTGAGGT	B102	ACCGCGAGGT
B57	GCCGTAAACG	B106	GCCTTCGGGT
B58	GTAACGGCTC	B107	TTGCCAGCAT
B59	CCGCAAGGAG	B108	GCTAACGCGT
B63	GAACGCTGGC	B109	ACGGTACCTG

The reference probe is designed to hybridise to all clones.

Following hybridisation each membrane was washed twice in 1×SSC buffer for 15 minutes. The wash conditions were identical for all probes. After washing, the arrays were allowed to dry completely, then taped to stiff paper to hold them flat and exposed directly to a phosphoimaging screen (Bio-Rad). The screens were scanned using a Personal Molecular Imager (Bio-Rad). Probes B39 and B74 were not included in the final analysis, as they did not produce useful signals due to an error made during the hybridisation process.

### Construction of Hybridisation Fingerprints from Array Data and Analysis

Analysis of the array information was performed as described by Bent et al. [22]. OFRG produces a hybridisation fingerprint for each clone by assigning a score of 0, 1 or N to each probe/clone combination according to whether the probe hybridised or did not hybridise to the clone. An example of a fingerprint obtained for a single clone using a combination of 35 probes is 0010000100001100110100010110N001001. These scores are generated by quantifying the intensity of spots on the arrays using suitable array analysis software. The quantified data are then transformed by defining threshold intensities for the assignment of positive (1), negative (0) and uncertain (N) scores according to the hybridisation patterns of the control clones, which have defined nucleotide sequences and theoretically calculated expected fingerprints.

Hybridisation data were quantified using Imagene v5.6 software (BioDiscovery Inc., Segundo, CA). Background values were subtracted from signal intensity values using customized software [22]. The background-subtracted signal intensities were then normalised by dividing the values obtained from the differential probes by the values obtained from the reference probe, which is designed to hybridise to all clones. The normalised intensity values were transformed into 1, 0 or N scores according to the threshold values determined using the 96 control clones as described by [22]. This process resulted in a fingerprint for each clone.

To place the clones into closely related taxonomic groups, fingerprints were clustered using the Greedy Clique Partition Package Tool (GCPAT) [25], which first resolves uncertain ‘N’ values and clusters identical fingerprints using the Unweighted Pair Group Method with Arithmetic mean (UPGMA) algorithm [26]. Since clusters consist of groups of clones with the same fingerprint, each cluster can be defined as an operational taxonomic unit (OTU) (equivalent to a phylotype or ‘species’).

After clustering, diversity levels in each sample were assessed by calculating the Simpson index of diversity (1-D = 1- Σn_i_(n_i_-1)/N(N-1)) where n_i_ was the number of clones in the *i*th OTU, and N was the total number of clones per sample. Comparisons of the overall structure of bacterial communities were made using principal components analysis. Because the total number of clones analysed for each sample varied slightly due to array printing errors (missing spots), the raw data consisting of the numbers of clones in each OTU was normalized by dividing by the total number of clones per sample. Only OTUs containing five or more clones were used in the analysis. Principal components analysis was performed using the correlation matrix in MINITAB version 15.1.30.0. Multi response permutation procedures (MRPP) analysis [27] was performed on the same normalized dataset using PC-ORD software [28] to test for significant differences in the overall distribution and abundance of phylotypes in each of the four sample categories.

Clusters consisting of six or more clones were subjected to indicator species analysis [27] in order to identify OTUs that contained significantly higher numbers of clones in each coral tissue category (H, M, D or C). OTUs that were significantly (p≤0.1) associated with one or more sample category were selected for identification by nucleotide sequence analysis. Clones from additional OTUs were also sequenced that were identified as highly abundant in one or more sample categories.

### 16S rRNA Gene Sequence Analysis

PCR conditions for the amplification of clone inserts for sequencing were the same as those used for amplification for macroarray construction (above). Sanger sequencing was performed on the amplified clone inserts with the forward primer 27F (5′ GAGCTCCAGAGTTTGATCMTGGCTCAG). Alignment and chimera checking was performed in Greengenes [29]. Chimera sequences were discarded from the final dataset. The sequences were then subjected to nucleotide-nucleotide BLAST searches to identify their nearest relatives in the GenBank database (http://www.ncbi.nlm.nih.gov/blast). Classification of isolates to genus level was achieved by submitting their 16S rRNA gene sequences to the Ribosomal Database Project (RDP) Classifier [30], which assigns a classification with at least 80% confidence based on known sequences contained in the RDP [31]. All clone sequences obtained were submitted to the NCBI database (accession numbers EU379566 and EU780226-EU780422, see Table S1).

### Isolation of Bacteria from Coral Samples

Coral fragments were placed into sterile mortars to which approximately 20 ml of autoclaved filtered seawater (FSW) was added. Each fragment was ground into a smooth slurry with a sterile pestle. In order to break up sticky aggregates of mucus and coral tissue, this slurry was vigorously passed several times in and out of a sterile 20 ml syringe first without a needle, and then with a 21 gauge needle attached. Four serial dilutions of 10^−1^ to 10^−4^ of the resulting slurry were prepared in sterile FSW. 10 mL aliquots of syringe samples were also diluted with sterile FSW. 100 µl aliquots of each dilution were spread on agar plates containing a non-selective marine heterotrophic growth medium (Salt Luria Broth SLB agar [32]), which were incubated at 30°C for up to five days. 30°C was chosen as the incubation temperature for bacterial cultures because this temperature resulted in relatively rapid growth of most bacterial strains, and the aim of this work was to isolate as many strains as possible as quickly as possible.

Inoculated plates were checked every twelve hours for the formation of bacterial colonies, as some colonies grew to observable size much faster than others. Colonies were differentiated based on their morphology (shape, size, texture and colour, growth rate) and semi-quantitative estimates of the total number of colony forming units (cfu) of each bacterial type on each plate were recorded after 48 hours incubation. Representatives of the most abundant colony types were subcultured on SLB agar plates. All isolates were subcultured at least twice on agar plates to ensure that cultures were axenic. Broth cultures were prepared by inoculating 5 ml Marine Yeast Tryptone (MYT) medium (0.8% w/v Bacto-tryptone, 0.5% w/v Bacto-yeast extract prepared in 75% v/v natural filtered seawater) with single colonies picked from pure subcultures grown overnight on SLB agar. Cultures were grown to stationary phase by shaking at 180 rpm at 30°C for 16 to 24 hours, (depending on the bacterial strain). Bacterial isolates were each given a unique name (e.g. 2.3.05 2MS3) which indicates the date on which the original sample was collected, and includes a number corresponding to the coral colony sampled, and letters indicating which section (apparently healthy tissue, margin of disease lesions, or dead coral skeleton) that isolate was obtained from.

### Phylogenetic Analyses of Culturable Bacteria Isolated from Corals

Cultured bacterial isolates that were numerically dominant on agar plates were identified by 16S rRNA gene sequence analysis. Genomic DNA was extracted from these cells with the Qiagen DNeasy blood and tissue kit (Qiagen-Hilden, Germany), using the protocol recommended for Gram-negative bacteria. 1 µl of bacterial genomic DNA extract was used as template for the PCR amplification of the 16S rRNA gene using the forward primer 27F (5′GAGCTCCAGAGTTTGATCMTGGCTCAG) and reverse primer 1492R (5′CACGYTACCTTGTTACGACTT) [33] The conditions for the PCR were the same as for the amplification of 16S rRNA genes from total coral DNA (above), except that the final extension step at 72°C was lengthened to 10 minutes to ensure that no truncated products were used for sequencing. PCR products were cleaned up using the Qiaquick spin PCR cleanup kit (Qiagen-Hilden, Germany), eluted in 10 mM Tris/HCl (pH 8.0) and quantified on agarose gels against 1kb standard DNA ladders (New England Biolabs). DNA sequencing, chimera checking, classification and comparison to online databases was performed as described above for the clones derived from the culture independent study.

Sequences obtained from cultured isolates were aligned against the complete 16S rRNA gene sequences of *E. coli* and representative type strains belonging to the classes *Gammaprotebacteria, Alphaproteobacteria* and *Bacteroidetes* taken from the RDP [31]. Alignment was performed in MEGA 5.0 [34] using the default ClustalW algorithm [35]. The aligned sequences were then cropped to produce an alignment of the 570 bp region corresponding to positions 130–700 of the *E. coli* 16S RNA gene. A phylogenetic tree was inferred in MEGA 5.0 [36] using the minimum evolution algorithm. A consensus tree inferred from 500 bootstrap replicates was used to determine the phylogenetic relationships of the isolates with each other and with the type strains. All sequences were submitted to the NCBI database (accession numbers EU267607-EU267670 and EU276972-EU277002, Table S2).

### Effects of Antibacterials on Disease Progression

The effect of antibacterial agents on the rate of ASWS lesion progression was tested to investigate the role of bacteria as causative agents of ASWS. Sections of several ASWS affected *T. mesenterina* colonies were collected and divided into fragments of approximately 15–30 cm^2^. These fragments were randomised, and no discrimination was made between fragments that were derived from different colonies. The position of the disease margin (M) at the interface between apparently healthy coral tissue (H) and dead coral skeleton (D) was marked by drilling a small hole (3 mm diameter). To ensure that only fragments with actively advancing disease lesions were used in the experiment, the fragments were placed in aquaria containing natural seawater maintained at 26°C for 48 hours prior to the addition of antibiotics. The fragments were maintained at 26°C because earlier experiments [10] had demonstrated that the disease progresses faster at this temperature. 26°C was also the maximum seawater temperature recorded in the field when high incidences of diseased corals were observed (Steven Dalton, personal communication). During this period the fragments were monitored for disease progression by measuring the distance between the disease margin and the reference hole. Fragments on which the disease margin did not progress at least 2 mm in 48 hours were not used for the experiment.

Twenty four fragments with actively progressing disease lesions were placed in separate 2.5 L plastic containers, each containing 2.0 L of filtered natural seawater at 26°C. The temperature experienced by the coral fragments was maintained at 25–26°C throughout the experiment and each container was separately aerated. The coral fragments were lit by four 40W 10,000K and four 40W actinic fluorescent globes for 12 hours per day.

Twelve of the 24 replicate coral fragments were randomly selected for treatment with antibiotics. Antibiotic stock solutions consisted of 100 mg ml^−1^ ampicillin in distilled water and 10 mg ml^−1^ tetracycline hydrochloride in 70% ethanol. The antibiotics were added to the water to give final concentrations of 25 ug ml^−1^ ampicillin and 10 ug ml^−1^ tetracycline. The remaining 12 fragments served as controls and were maintained in filtered seawater without any additions. Every 48 hours the coral fragments were removed from the containers and the progression of the disease lesion was determined by measuring the distance from the reference hole to the disease margin. The fragments were then transferred to clean containers containing fresh filtered seawater at 26°C, and fresh antibiotics were added to the treated fragments. The experiment was continued in this manner for 12 days.

Prior to statistical analysis, disease progression measurements were converted to overall rates of progression in mm day^−1^ by dividing the total distance the lesion had progressed by the time since the addition of antibacterials. Data were tested for normality using the Anderson-Darling test and for homogeneity of variance using Levene’s test. The data was transformed to correct for non-normal distribution. This was done by adding a constant of 1 to all lesion progression rates to eliminate zero values, then calculating the inverse square root of the resulting value. Repeated measures ANOVA was used to test for differences in the rates of disease progression between the treatments and controls. All analyses were performed using MINITAB version 15.1.30.0.

## Results

### Culture Independent Analysis of Bacterial Communities Associated with ASWS using the ORFG Method

A total of 8094 fingerprints were obtained from the array of 9600 clones following removal of positions in the array that did not yield sufficient hybridization data for analysis. GCPAT analysis of these fingerprints grouped them into 3208 clusters of clones with identical fingerprints. Nucleotide sequence identity within clusters ranged from 90.0% to 100.0%, with a mean identity level of 97.9% (data not shown). It has been previously demonstrated that the OFRG method is able to discriminate between 16S rRNA genes at the 97% identity level or better [37]. The clusters generated can be considered operational taxonomic units (OTUs) or phylotypes. Of these 3208 phylotypes, 201 included six or more clones, and 113 of these (representing a total of 2991 clones) were selected for identification by sequence analysis. (see Table S1 for a full list of sequenced clones). Of the 113 phylotypes identified by sequence analysis, 11 were significantly (indicator species analysis p≤0.1) more abundant in the active margin of disease lesions (M), and 7 of these phylotypes were present in all M samples and absent from all unaffected control (C) samples.

**Figure 2:**
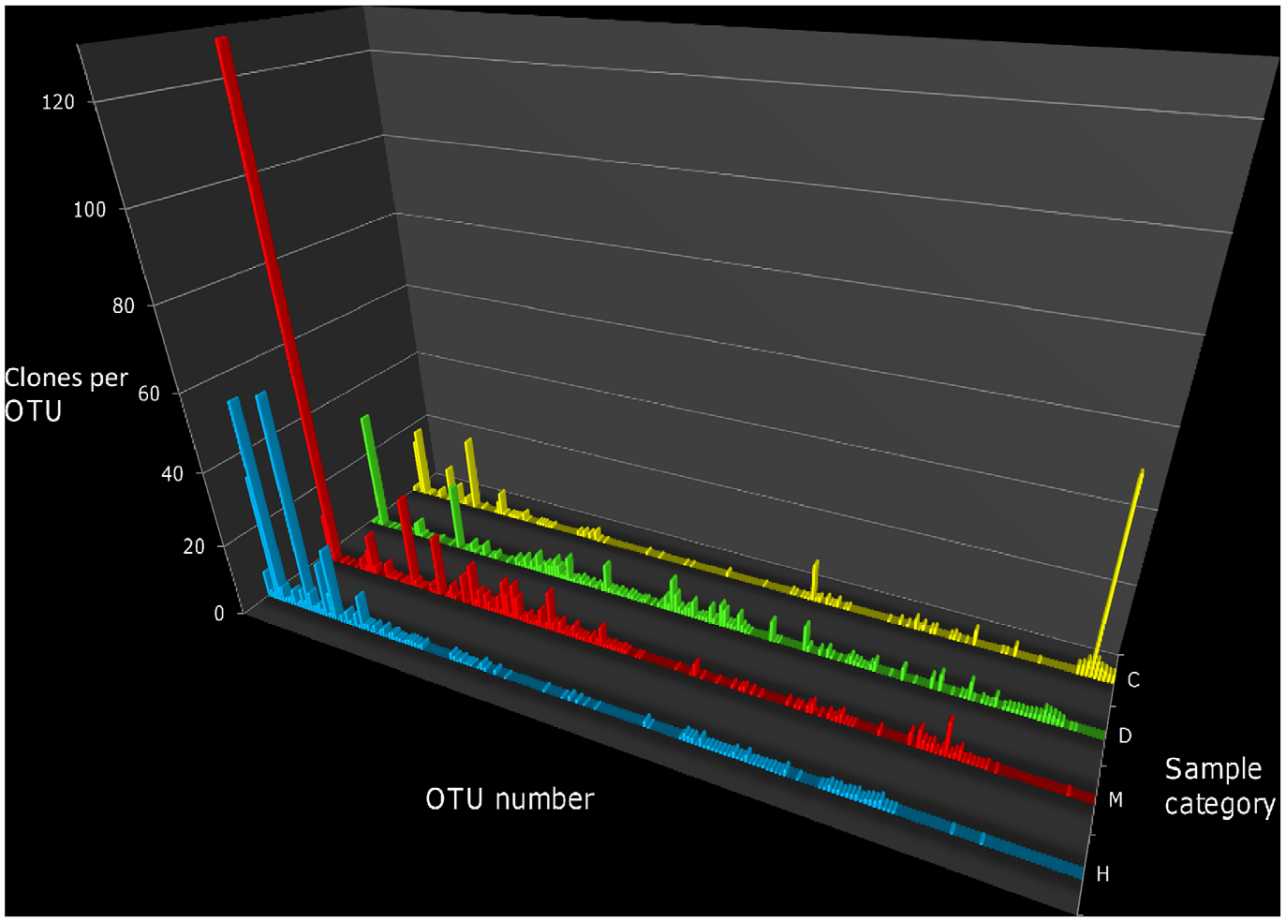
Comparative profiles of the bacterial communities associated with healthy and ASWS affected coral colonies. H - apparently healthy tissues of ASWS affected *T. mesenterina* colonies, M - the margin of disease lesions, D - exposed skeleton adjacent to the margin and C - healthy control colonies with no signs of ASWS. ‘OTU number’ refers to the number each OTU was assigned by the GCPAT clustering software. The bar at each position on the x-axis represents an OTU consisting of clones with identical hybridisation fingerprints. The height of the bars represents the mean number of clones per OTU for all replicate samples from each sample category. Only OTU’s containing ≥5 clones are shown.

**Figure 3:**
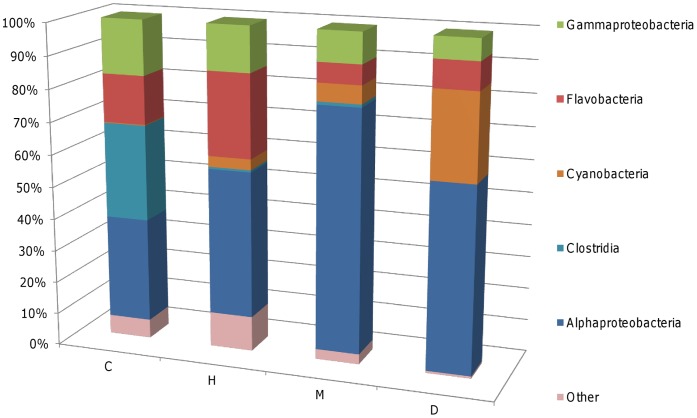
Relative proportions of major bacterial taxa in sequenced OTUs from each sample category. H - apparently healthy tissues of ASWS affected *T. mesenterina* colonies, M - the margin of disease lesions, D - exposed skeleton adjacent to the margin and C - healthy control colonies. Stacked bars were calculated from the mean numbers of clones belonging to each taxa in each sample category. Only OTUs identified by 16S rRNA gene sequence analysis are presented.

**Figure 4:**
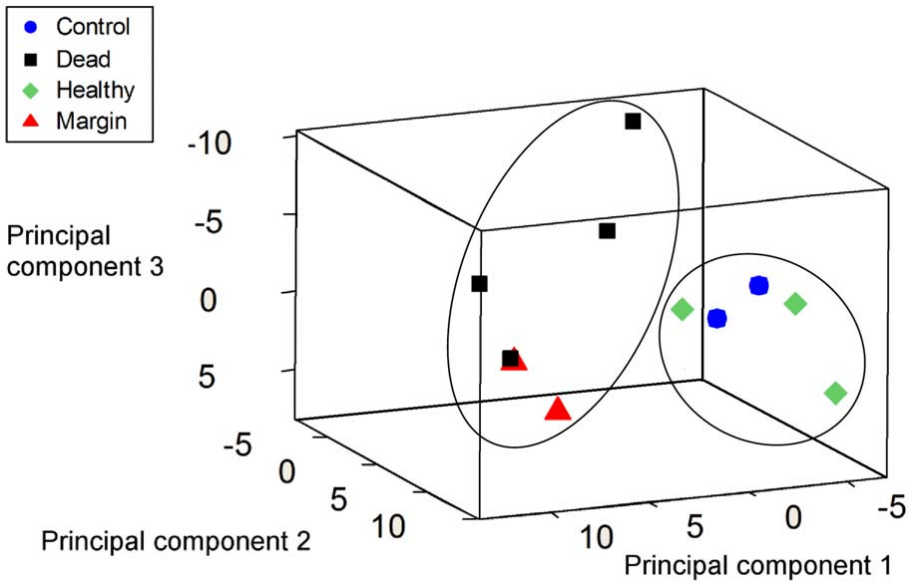
Three dimensional principal component plot of OFRG data. Plot represents the first three principal components, which represent 52% of the variability in the data (Table 4). The samples grouped into two clusters (indicated by circles), consisting of a ‘healthy tissue’ cluster, and a ‘disease lesion’ cluster. These clusters were also supported by MRPP analysis. H - apparently healthy tissues of ASWS affected *T. mesenterina* colonies, M - the margin of disease lesions, D - exposed skeleton adjacent to the margin and C - healthy control colonies.

Figures 2 and 3 illustrate the overall structure of the bacterial communities associated with each sample category. Community diversity was very high for all sample categories (Simpson’s index (1-D)>0.9). Four way comparison of each sample category (H,M,D and C) by MRPP analysis indicated that there were significant differences between categories in the overall structure of the bacterial communities (T = −1.898, p = 0.04). The most pronounced differences were observed between living coral tissues (sample categories H and C) and sections of ASWS affected colonies covered by disease lesions (sample categories D and M) (T = −4.271, p = 0.001). No significant difference was observed between the overall structure of the bacterial communities associated with the living tissue of apparently healthy coral colonies (sample category C) and that of communities associated with living tissue of ASWS affected colonies (sample category H) (T = −0.101, p = 0.41). These results are supported by principal component analysis which indicates that the bacterial communities grouped into two clusters which contained live coral tissues (categories H and C) and diseased/dead coral tissues (categories D and M) (Figure 4).

### Identities of Selected Phylotypes

BLAST analyses confirmed that the majority of the bacterial clones obtained from all of the coral samples were of marine origin (Table S1). 43 of the 113 phylotypes selected for 16S sequencing and BLAST analysis were identified as members of the alphaproteobacteria division. These phylotypes contained 54.2% of the 2991 clones represented by the 113 OTUs selected for sequencing. Other bacterial taxa represented included Flavobacteria (13.8% of clones), Gammaproteobacteria (12.1%), Cyanobacteria (10.6%), Clostrida (4.4%), Betaproteobacteria (1.3%), Chlorobia (1.1%), Bacteroidetes (1.1%) and Epsilonproteobacteria (0.4%). The classes Planctomycetes, Spirochaetes, and Verrucomicrobiae each comprised 0.2% of the clones represented by the sequenced OTUs (Figure 3).

Indicator species analysis identified a number phylotypes which were significantly more abundant in certain sample categories (Tables 2 and 3) Of particular interest are OTUs 530 and 534 which exhibited high levels of sequence similarity (≥97%) to *Rhodobactereaceae* species associated with juvenile oyster disease [38] and black band disease in corals [39], respectively. Both of these phylotypes were present in all samples of the active margin of disease lesions (M) and absent from samples of unaffected control colonies (C). OTU 530 was closely related (97% sequence identity) to *Roseovarius crassostreae*, the aetiological agent of juvenile oyster disease [38], [40], [41]. Bacteria related to this pathogen have also been detected in association with corals affected by a number of other disease conditions [39], [42], [43].

**Table 2:** Operational taxonomic units significantly associated with coral disease lesions.

OTU	Acession number(s)	Indicator for category*	p-value	Nearest relative (with source where available) [accession number]	% Identityto nearest relative	RDP classifier taxon (80% confidence threshold)
806	EU780329 EU780328	D	0.0232	Uncultured bacterium (marine microbialmat) [GQ441254]	99	Chloroplast of *Bacillariophyta* (diatom)
265	EU780267 EU780268	M/D	0.0478	Uncultured bacterium (marine sponge) [AY845242]	98	*Rhodobactereaceae* (Genus *Roseobacter*)
210	EU780230 EU780229	M	0.0076	*Thalassobius mediterraneus* [AJ878874]	99	*Rhodobactereaceae* (unclassified)
329	EU780228 EU780227	M	0.0110	Rhodobacteraceae bacterium [AY177714]	98	*Rhodobactereaceae* (Genus *Roseovarius*)
449	EU780234 EU780233	M	0.0196	Uncultured alpha proteobacterium (squid) [AJ633989]	96	*Rhodobactereaceae* (unclassified)
452	EU780244 EU780243	M	0.0530	Uncultured bacterium (seafloor sediment) [AY35417]	90	Unclassified Gammaproteobacteria
459	EU780246 EU780245	M	0.0498	Uncultured epsilon proteobacterium(deep sea isolate) [AB113178]	90	Unclassified Bacteria
480	EU780232 EU780231	M	0.0204	*Thalassobius mediterraneus* [AJ878874]	99	*Rhodobactereaceae* (unclassified)
521	EU780240 EU780239	M	0.0494	Uncultured bacterium (from sponge)[AY845242]	98	*Rhodobactereaceae* (Genus *Roseovarius*)
530	EU780236 EU780235	M	0.0170	*Roseovarius crassostereae* [AF114484](associated with juvenile oyster disease)	97	*Rhodobactereaceae* (Genus *Jannaschia)*
534	EU780260 EU780259	M	0.0254	Uncultured alpha proteobacterium (associatedwith black band disease in corals) [AF473915]	97	*Rhodobactereaceae* (unclassified)
616	EU780248 EU780247	M	0.0224	*Thiothrix* sp. (marine isolate) [DQ067608]	96	*Thiotrichaceae* (Genus *Thiotrix*)

* Sample categories - C - Apparently healthy coral colonies with no sign of ASWS, H - Apparently healthy tissue of colonies affected by ASWS, M - Margin of ASWS disease lesions, D - Exposed coral skeleton adjacent to margin of disease lesion.

**Table 3:** Operational taxonomic units significantly associated with living, apparently healthy coral tissues.

OTU	Acession number(s)	Indicator for category*	p-value	Nearest relative (with source whereavailable) [accession number]	% Identityto nearest relative	RDP classifier taxon (80% confidence threshold)
201	EU780300 EU780299	H/C	0.0046	Uncultured bacterium (*Montastrea franksi*(coral)) [GU118721]	84	*Flavobacteriaceae* (unclassified)
320	EU780292 EU780291	H/C	0.0406	*Comamonas* sp. M2T2B14 (mixture of bovinedung and urine, cow's milk, and yogurt undergoingaerobic fermentation) [GQ246691]	99	Comamonadaceae (genus Comamonas)
348	EU780294 EU780293	H/C	0.0144	Uncultured bacterium (wound of mouse skin) [HM820111]	99	Comamonadaceae (genus Comamonas)
390	EU780295	H/C	0.0144	Uncultured bacterium [HM186874]	99	Xanthomonadaceae (genus Stenotrophomonas)
406	EU780319	H/C	0.0160	Uncultured bacterium (*Montastrea franksi* (coral)) [GU118721]	84	Flavobacteriaceae (unclassified)
441	EU780296	H/C	0.0026	Uncultured bacterium (Human intestinal pouch) [GQ157102]	99	Pseudomonadaceae (genus Pseudomonas)
236	EU780304 EU780303	H/C	0.0070	*Stenotrophomonas maltophilia* strain CCUG 50297[HQ434487]	99	*Xanthomonadaceae* (genus *Stenotrophomonas*)

* Sample categories - C - Apparently healthy coral colonies with no sign of ASWS, H - Apparently healthy tissue of colonies affected by ASWS, M - Margin of ASWS disease lesions, D - Exposed coral skeleton adjacent to margin of disease lesion.

**Table 4:** Results of principal components analysis of the data generated by the culture-independent study.

Principal component	Eigenvalue	Proportion of variabilityrepresented	Cumulative proportion of variability represented
1	31.95	0.21	0.21
2	25.43	0.16	0.37
3	23.38	0.15	0.52
4	15.48	0.10	0.62
5	13.71	0.09	0.71
6	11.50	0.07	0.78
7	10.30	0.07	0.85
8	9.23	0.06	0.91
9	8.02	0.05	0.96
10	5.99	0.04	1.00

Also of interest is OTU 616, which was present only in samples of the active margin of disease lesions (M) and dead coral skeleton (D). BLAST analysis indicates that this phylotype is closely related (96% 16S rRNA sequence similarity) to filamentous sulphur oxidising bacteria in the genus *Thiothrix*. *Thiothrix* has been implicated in mortality of lobster larvae in aquaculture systems [44].

A number of phylotypes were identified by indicator species analysis that were significantly more abundant in samples of living coral tissue (sample categories H and C) than in samples of the margin of ASWS lesions (sample category M) or dead coral skeleton (category D). The most noteworthy of these were OTUs 201 and 236 (Table 3), which belonged to the families *Flavobacteriaceae* and *Xanthomonadaceae* respectively. One phylotype (OTU 2921) was also noted that was not detected by indicator species analysis, but was relatively abundant in only one of the two replicate samples of apparently healthy coral (sample category C), and absent from all other samples. BLAST analysis indicated that this phylotype was closely related (99% sequence identity) to an uncultured *Clostridiaceae* bacterium from rainbow trout gut (Table S1). This abundance of this phylotype is the reason for the apparently high proportion of *Clostridia* in sample category ‘C’ (Figure 3).

### Culturable Bacteria Associated with ASWS of *T. mesenterina*

**Figure 5:**
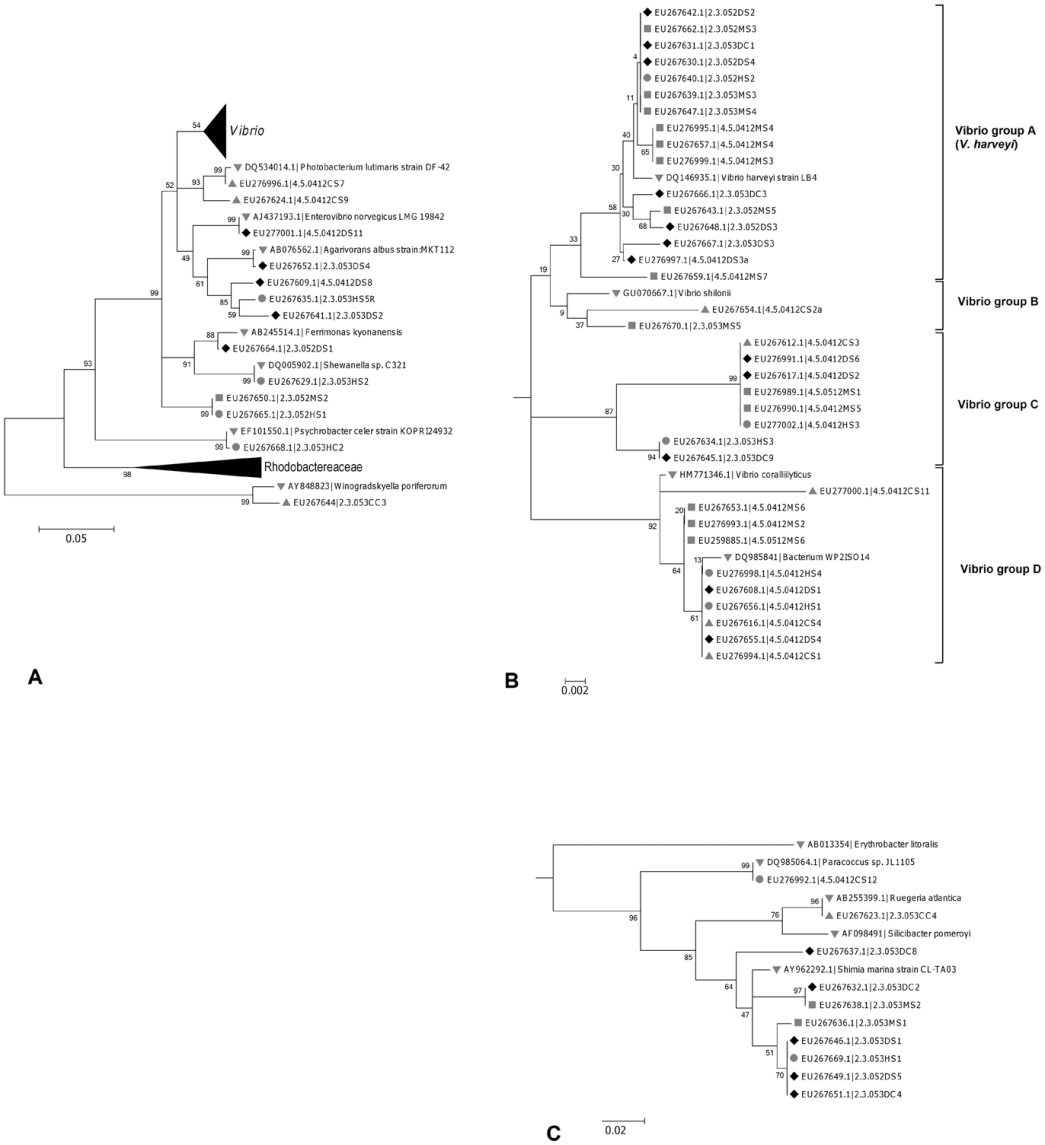
Phylogenetic tree showing the taxonomic relationships of culturable bacterial isolates. **A.** All isolates. **B.** *Vibrio* spp. subtree **C.** *Rhodobactereacea* subtree. Phylogeny was inferred using the maximum likelihood method based on the Tamura-Nei model [66]. The tree displayed is the bootstrap consensus tree inferred from 500 replicates. Branches corresponding to partitions reproduced in less than 50% bootstrap replicates are collapsed. The percentage of replicate trees in which the associated taxa clustered together in the bootstrap test (500 replicates) are shown next to the branches. The level of bootstrap support near the ends of some branches is low because these branches included several isolates with very high levels of sequence identity. All positions containing gaps and missing data were eliminated, leaving a total of 466 positions in the final dataset. Phylogenetic analyses were conducted in MEGA4 [34]. The evolutionary distances were computed using the Maximum Composite Likelihood method. The trees are drawn to scale, with the scale bars indicating the number of substitutions per nucleotide position. Clusters of organisms at branch ends share >99.5% sequence similarity, and are treated as the same organism for the purposes of this discussion. Markers on the left indicate the sample categories that each organism in the tree was isolated from (♦ exposed dead skeleton adjacent to living tissue of ASWS affected colonies (D), ▪ the interface between exposed skeleton and apparently healthy tissue at the margin of disease lesions (M), • the apparently healthy tissue adjacent to disease lesions on ASWS affected colonies (H), ▴ Apparently healthy colonies(C), ▾ related sequences retrieved from public databases).

A total of 60 strains of numerically dominant culturable bacteria were isolated from all sample categories and identified by 16S ribosomal gene sequencing (Table S2). These 60 isolates included 11 from apparently healthy coral colonies with no sign of ASWS (C), 10 from the apparently healthy tissues of ASWS affected colonies (H), 15 from the margin of active disease lesions (M), and 24 from recently denuded skeleton adjacent to the margin of disease lesions (D). Phylogenetic analyses indicated that of these 60 isolates, 49 (82%) belonged to the class *Gammaproteobacteria*. All of the remaining isolates belonged to the class *Alphaproteobacteria* with the exception of a single strain isolated from a apparently healthy coral colony (2.3.053CC3), that belonged to the family *Flavobacteriaceae* in the class *Bacteroidetes*. Figure 5a illustrates the phylogenetic relationships of these isolates with each other and with ribosomal RNA gene sequences of related organisms. The level of bootstrap support near the ends of some branches is low because these branches included several isolates with very high levels of sequence identity. Many isolates formed clusters of very closely related (>99% sequence identity) or identical 16S ribosomal gene sequences (Figures 5b and 5c). It is evident from these clusters that the same bacterial species were isolated multiple times from different samples, and sometimes more than once from the same sample. Each of these clusters is considered a distinct ribotype for the purposes of this discussion. Identical ribotypes were frequently isolated from both coral mucus and coral tissues. Indeed, since no discernible differences were observed in the culturable bacterial communities derived from coral mucus and coral tissues, no further distinction will be made between them.

39 of the 60 bacterial strains identified by 16S gene sequencing (65%) were members of the family *Vibrionaceae*. These isolates may be broadly divided into four groups on the basis of their phylogeny. These groups are designated *Vibrio* groups A–D (Figure 5b): *Vibrio* group A contained 14 bacterial isolates which all shared >97% sequence similarity. This group of isolates is of particular interest because it consists entirely of strains which originated from ASWS affected *T. mesenterina* colonies. BLAST analysis indicates that these strains were closely related to pathogenic *V. harveyi* strains from fish and strains associated with mortality of lobsters grown in aquaculture (accession numbers DQ314530.1, DQ314529.1, DQ831087.1, DQ831116.1 and DQ831086.1). Of the 14 isolates in this monophyletic group, only one was obtained from the apparently healthy, living tissue of an ASWS affected colony, while the rest were all isolated from either the actively advancing margin of disease lesions, or the denuded coral skeleton adjacent to the disease margin. No *V. harveyi* strains were isolated from apparently healthy coral colonies.

*Vibrio* groups B, C and D included the remaining *Vibrio* isolates, which were more diverse than the *V*. *harvey*i group. Phylogenetic analysis grouped them into three distinct clusters with other *Vibrio* species found in seawater or associated with coral and other marine organisms (Figure 5b). Noteworthy isolates in this group included 4.5.0412CS1, which was isolated from a healthy coral colony. Although the nearest relative of this isolate identified by BLAST analysis was an uncultured bacterium from seawater, the next closest relative, with 96% sequence similarity, was an unclassified *Vibrio* strain known to inhibit pathogens in mollusks (accession number AY034144.1). Other isolates of interest included 2.3.053HS3 and 2.3.053DC9 which were respectively isolated from apparently healthy (H) and dead (D) areas of the same ASWS affected coral colony. These isolates both exhibited >97% sequence similarity to *Vibrio* sp. HB-8 (accession number AY876051.1), which is associated with brown band disease in *Acropora muricata* on the Great Barrier Reef. Three strains (4.5.0412MS7, 11.5.052CC11, 11.5.052CS2) were also isolated from both the active margin of ASWS lesions and from apparently healthy coral colonies that shared >97% sequence similarity with *Vibrio shiloi*, the causative agent of bacterial bleaching of the coral *Oculina patagonica* [45].

Of the 16 sequenced isolates classified by the RDP as Alphaproteobacteria, 13 (81%) belonged to the *Rhodobacteraceae* family. On the basis of semi-quantitative counts of colony forming units, *Rhodobactereaceae* species were the second most numerically dominant group of bacteria (after the *Vibrionaceae*) isolated from ASWS affected corals. With the exception of the isolate 2.3.053DC8, all of the *Rhodobactereaceae* spp. isolated from diseased corals were closely related to *Shimia marina* (Figure 5c), a bacterium isolated from fish farm biofilms [46]. No *Rhodobactereaceae* strains were isolated from apparently healthy *T. mesenterina* colonies.

Most of the remaining isolates classified within the gammaproteobacteria and did not appear to be specifically associated with any sample category, with the possible exception of two isolates originating from the same apparently healthy coral (4.5.0412CS9 and 4.5.0412CS7). These isolates were closely related (97–99% sequence identity) to *Photobacterium* species, and produced luminescent colonies on SLB agar. No *Photobacterium* strains were isolated from any part of *T. mesenterina* colonies affected by disease. The remaining isolates were diverse and included representatives of the families *Moraxellaceae*, *Shewanellaceae*, *Alteromonadaceae*, *Ferrimonidaceae*, *Pseudoalteromonadaceae*, and *Pseudomonadaceae.* Two isolates with 100% sequence identity (2.3.052MS2 and 2.3.052HS1) represent a single organism that was unrelated to described families within the gammaproteobacteria and was designated ‘unclassified’ by the RDP [30]. Isolate 2.3.053DS4 originated from the dead skeleton of an ASWS affected colony and was classified as belonging to the genus *Agarivorans*, which falls amongst genera of uncertain status in incertae sedis 7 of the *Alteromonadales*. The ability of this strain to degrade agar suggests that it is likely to be an algae associated species.

It is interesting to note that a large proportion (30–50%) of colony forming bacteria that grew in mixed culture when the original serial dilutions of H and C samples were first plated on SLB agar did not survive in axenic culture. It is possible that they represented fastidious or syntrophic organisms that comprised large proportions of the communities associated with living coral tissue. In contrast, a much lower proportion (approximately 5–10%) of colony forming bacteria from the M and D sample categories consisted of strains that failed to grow in axenic culture.

### Effect of Antibacterials on Disease Progression

**Figure 6:**
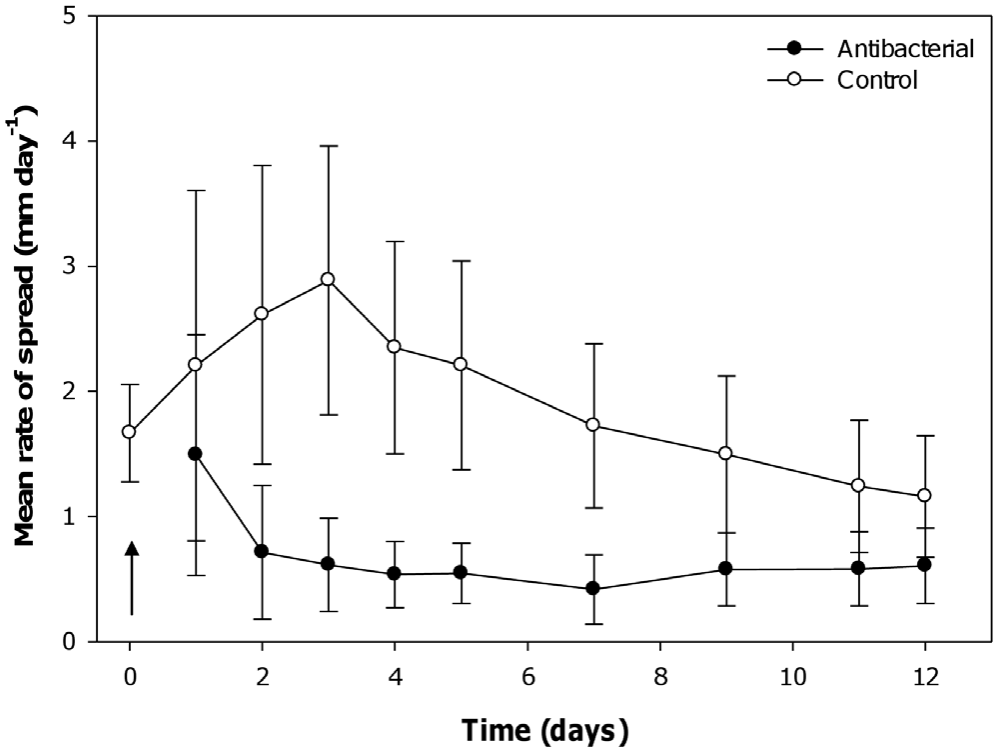
Effect of antibacterials on rate of disease progression. Mean rate of spread of disease lesions (± standard errors) on *T. mesenterina* fragments maintained at 26°C in the presence and absence of antibacterials. Both control and treatment fragments were maintained for two days before the addition of antibacterials (indicated by arrow).

Repeated measures ANOVA showed a significant effect of the antibacterial treatment on the rate of disease lesion progression (F = 7.71, p = 0.006). This result indicates that bacterial infection is likely an important factor in the progression of ASWS lesions. Initially, the addition of antibacterials to disease affected *T. mesenterina* fragments resulted in a reduction of the mean rate of disease progression from 1.3 mm day^−1^ to 0.7 mm day^−1^ within 48 hours of the commencement of the experiment (Figure 6). This reduction in rate of spread continued for the first seven days following the addition of antibacterials. In contrast, the disease lesions on the untreated control fragments progressed at an accelerating mean rate for the first three days of the experiment and remained high before slowing to a rate similar to that observed in the treated fragments (Figure 6). By the end of the experiment, disease progression had halted in 57% of the fragments treated with antibiotics, and 44% of the control fragments.

## Discussion

### Australian Subtropical White Syndrome is likely to have a Bacterial Aetiology

The culture based and culture independent studies presented here demonstrate that there is a distinct difference between the structure of the bacterial communities associated with the apparently healthy tissues of *T. mesenterina* and those associated with ASWS lesions. The overall pattern of the shift in the composition of bacterial communities from the living tissue of infected colonies, to the advancing disease margin and exposed dead skeleton is consistent with previously reported observations of the bacterial communities associated with other coral diseases such as black band disease, white band disease and white plague [42], [43], [47]. The observation of differences in bacterial community structure between apparently healthy and diseased affected parts of the coral was not surprising. Death of the coral tissue will drastically alter the microenvironment available for bacterial colonization, and it is likely that opportunistic heterotrophs that can take advantage of the nutrients available in the decaying coral tissue will become dominant in disease lesions. The important question is whether the distinct bacterial community associated ASWS lesions is present as a consequence of the disease, or the disease is present as a consequence of infection with bacterial pathogens. The fact that the rate of progression of ASWS lesions was significantly inhibited by treatment with broad-spectrum antibacterial compounds strongly suggests that at least some component of the ASWS associated bacterial community may be pathogenic.

When the results of this study are considered in the light of the results of previous studies which have made it very clear that ASWS in *T. mesenterina* is an infectious disease caused by a transmissible biotic agent or agents [10], [11], a bacterial aetiology seems likely. However, the identity of the specific bacterial pathogen remains unclear. It should be noted that viruses associated with corals are also poorly understood [6], [48] and therefore the role of viral infection in this coral disease cannot be excluded.

The overall composition of the bacterial communities associated with the apparently healthy tissue of ASWS affected *T. mesenterina* colonies and the communities associated with the tissues of apparently healthy colonies appeared to be similar. This suggests that in the case of ASWS, disease signs do not develop as the result of a major perturbation of the normal microbiota associated with the coral colony. This mechanism for development of some coral diseases has been suggested by Kline [49]. Rather, it is more likely that ASWS is the result of infection with a specific bacterial pathogen. The fact that few differences were observed between the bacterial communities associated with healthy *T. mesenterina* colonies and the apparently healthy tissues of ASWS affected colonies indicates that any bacterial pathogens responsible for causing the disease are only present in the active disease lesions, and not in the unaffected tissues. This suggests that the pathogen must be introduced to the coral colony from an external source. Previous work has provided some evidence that a pathogen may be introduced to *T. mesenterina* by a predatory nudibranch (*Phestilla* sp.), which was observed to cause lesions through its feeding activity that continued to expand even after the nudibranch was removed [11].

### Bacterial Communities Shifts Associated with Healthy and ASWS Affected *T. mesenterina*

The results of the culture independent study suggest that the bacterial communities associated with both healthy and diseased *T. mesenterina* are dominated by members of the division alphaproteobacteria. This finding contrasts with the results of the culture based survey, which suggested that the bacterial communities of both healthy and ASWS affected *T. mesenterina* were dominated by gammaproteobacteria, in particular *Vibrio* spp. The OFRG analysis indicated that members of the gammaproteobacteria represented only a small proportion of phylotypes that correlated with the disease affected corals, and only one OTU, comprising just six clones, was identified as belonging to the *Vibrionaceae*. It is unlikely that the failure to detect large numbers of *Vibrio* species was due to bias in the PCR amplification of 16S genes from the coral samples, as the same PCR primers used in this study are effective in amplifying 16S rRNA genes from cultured *Vibrio* isolates. On the contrary the apparent dominance of *Vibrio* species in the culture-based study was almost certainly due to biases inherent in the media and conditions used for isolation of bacteria. This result is not surprising when it is compared to previously reported studies. Koren and Rosenberg [50] showed that *Vibrio* spp. comprised only 1% of the cloned bacterial 16S genes recovered from the coral *Oculina patagonica*, and that only 0.1–1% of the total coral-associated bacteria were culturable. It is also possible that the slightly elevated incubation temperature of 30°C, which was chosen to increase the growth rate of culturable bacteria, introduced some bias towards the growth of *Vibrio* spp., which is often favoured at higher temperatures [51], [52]. Groups including *Vibrio* spp. may also have been enriched during sample handling and transport, as the samples were transported in seawater at ambient temperature, rather than on ice. The bias towards *Vibrio* spp. in the culture-based study highlights the importance of using both culture-based and culture-independent studies in efforts to characterize microbial community responses to environmental changes or identify unknown pathogens from environmental samples.

Although the overall composition of the bacterial communities associated with the apparently healthy tissue of ASWS affected colonies was similar to the community associated with the colonies with no sign of ASWS, there were some exceptions, particularly amongst the culturable bacteria. Members of the genera *Photobacterium* and *Paracoccus* were isolated only from apparently healthy *T. mesenterina* colonies, and were not detected at all in any disease affected category using either the culture based or culture independent approach, while members of the genera *Psychrobacter* and *Shewanella* were isolated only from the apparently healthy tissue of ASWS affected colonies and were not detected in any other sample category. These observations suggest that a subtle shift in the bacterial community composition may precede the development of disease characteristics, or may indicate a whole-colony integrated physiological response to the disease lesion, which in turn results in small shifts in the composition of the associated bacterial community. Such a response would be consistent with the whole-colony response to mechanical lesions observed by Oren et al. [53]. Changes in the bacterial community associated with the apparently healthy sections of disease affected coral colonies have been described for other coral disease states such as plague like disease of *Montastrea annularis* [42], and type I white band disease of *Acropora palmata* [43]. Pantos et al. [43] proposed a number of explanations for the whole-colony effect, including that the stress of the disease lesion causes a reduction in production of antimicrobial compounds by the coral host, or otherwise induces some alteration of the host physiology which results in changed conditions for the associated bacterial community.

It has been previously demonstrated that it is possible to halt the progression of ASWS through mechanical removal of the actively advancing margin of disease lesions [10]. This suggests that the causative agent of ASWS is not present in the apparently healthy tissue of disease affected colonies. It follows that if candidate pathogens are to be identified they should be found in microbiological samples taken from the disease margin (M) or the dead skeleton (D) of ASWS affected colonies. The high level of bacterial diversity in these sample categories and lack of an obvious dominant organism makes it difficult to identify a specific pathogen, however some candidates were identified in this work.

### Potential Pathogens Identified in this Study

The phylogenetic data obtained from both the culture-based and culture independent studies allowed inferences to be made that particular bacterial strains or OTUS could potentially be causative agents of ASWS. Bacteria that were strongly associated with ASWS affected *T. mesenterina* colonies, and which shared high levels of 16S rRNA gene sequence similarity with known pathogens or bacteria associated with other coral diseases were identified as potential pathogens.

In the culture-independent study, the presence of ribotypes with high degrees of similarity to *Roseovarius crassostereae*, the causative agent of juvenile oyster disease, is consistent with the findings reported by authors investigating the bacterial communities associated with corals affected by white band disease [42], black band disease [39], [54] and a plague-like disease [43]. These bacteria, which belong to the diverse and cosmopolitan *Roseobacter* clade, were present in all samples from the margin of the disease lesion (M), and were not detected in samples from unaffected control colonies (C). The fact that these bacteria are associated with a range of different types of disease lesions, on a range of different coral species suggests that they may be present simply as opportunists, and are not directly responsible for causing the disease. The possible role of these bacteria as pathogens cannot be ruled out, however. Although attempts to isolate them in culture have thus far been unsuccessful (data not shown), future work should focus on enriching for *Roseovarius* so that they may used in infection experiments to attempt to fulfill Koch’s postulates.

The identification of a phylotype in association with ASWS affected tissues that was closely related to the filamentous sulphide oxidising bacterium *Thiothrix* is also interesting. *T. mesenterina* fragments that became infected with ASWS under laboratory conditions via contact with either other infected fragments or contact with a predatory nudibranch were often observed to become covered with a dense mat of white bacterial filaments [10], [11]. Based on its general morphology and the presence of visible sulphur granules, this filamentous organism had previously been identified as a *Beggiatoa* species. *Thiothrix* is closely related to *Beggiatoa* and has a very similar morphology and ecological role as a sulphide oxidiser. Given that no *Beggiatoa* sequences were detected in the OFRG study, it is considered highly likely that the filamentous bacterium that was previously observed in association with ASWS was actually *Thiothrix*. This genus has been implicated in the mortality of phyllosoma larvae of tropical rock lobsters in aquaculture systems, where it has been observed to cause fouling of its host’s appendages during mass mortality events [44], [55]. Filamentous sulphide oxidising bacteria (*Beggiatoa* spp.) have also been reported in association with black band disease of corals [56], [57], but are not present in all cases of the disease [39], [58]. Based on these reports it seems most likely that *Thiothrix* appears as an opportunist that takes advantage of the sulphide that is likely released from the decaying coral tissue at the margin ASWS lesions.

In the culture-based study, the fourteen isolates that were closely related to pathogenic *Vibrio harveyi* strains (Figure 5b) represent a single monophyletic group which was detected in the ‘D’ and ‘M’ samples of all three of the ASWS affected colonies sampled. It was also detected in the apparently healthy tissues (H) of two of the same three affected colonies, but was not observed in any healthy colonies (C). The strong association of this strain with ASWS affected corals suggests that it may play a role as a pathogen. *V. harveyi* is well known as a pathogen of wide range of marine organisms, including bony fish, sharks, prawns, holothurians, lobsters and abalone, and has been reported as causing opportunistic infections in humans [59]. *V. harveyi* strains have also been reported in association with diseased corals. Ritchie and Smith [60] observed a strong association of *V. charchariae* (a junior synonym of *V. harveyi*) with WBDII in *Acropora cervicornis* and proposed that it may be the causative agent responsible for the disease. More recently, Gil-Agudelo et al. [18] reported preliminary evidence from field infection trials to suggest that this may indeed be the case. *V. harveyi* has also been reported in association with rapid tissue necrosis of *Pocillopora damicornis* [61], and yellow band disease of Caribbean and Indo-Pacific corals [62]. Considering its wide host range it is possible that *V. harveyi* is simply an opportunistic pathogen of stressed or immunocompromised marine organisms. The potential role of this bacterium in ASWS pathogenesis should be investigated further.

An alphaproteobacterium closely related to *Shimia marina* was also observed in high numbers only in ASWS affected *T. mesenterina* colonies and was not detected in healthy controls. Other members of the family *Rhodobacteraceae* are known to cause disease in oysters (Boettcher et al. 2005). The role of this bacterium in ASWS warrants further investigation, however as this strain was only isolated from two of the three diseased colonies sampled it does not seem to be as strong a candidate as the *Vibrio* isolate, which was identified in all disease samples.

In order to establish whether any of these putative causative agents of ASWS are actually pathogenic to corals, or are simply present as opportunistic heterotrophs in the ASWS lesion community, their ability to induce the signs of ASWS must be verified experimentally. Preliminary infection trials using cultures of the *Vibrio harveyi* and *Shimia* spp. strains isolated in the culture-based study inoculated into the water column of aquaria containing healthy *T. mesenterina* did not result in disease (data not shown). However it is now understood that direct contact with diseased coral tissue, or contact with a vector carrying the causative agent is required for transmission of ASWS from one colony to another [10], [11]. It is possible that infection trials using a different method of inoculation would produce different results.

### Coral Probiotic Bacteria

It is possible that some of the bacteria that were strongly associated with healthy coral tissues are involved in defending the coral against infection. Screening of the cultured isolates obtained here for antagonistic activity against known pathogens of marine organisms is currently underway. Several strains of *Vibrio, Pseudoalteromonas* and *Psychromonas* have been identified thus far that are able to inhibit the growth of a *Vibrio* species pathogenic to lobster in *in vitro* assays (L. Pereg, unpublished).

A dominant phylotype was identified in the culture independent study (OTU 201) that was very strongly associated with both apparently healthy coral colonies and the living, apparently healthy tissue of ASWS affected colonies. This phylotype shared only 84% 16S rRNA gene sequence similarity with its nearest relative, which was an uncultured bacterium associated with the Caribbean coral *Montastrea annularis*. It is difficult to speculate about the role of this phylotype in the health of the coral host without more information regarding its identity and biological function. A second phylotype identified in strong association with living coral tissue (OTU 236) shared 99% 16S rRNA gene sequence similarity with *Stenotrophomonas maltophilia*. This organism is a recognised pathogen of humans, which is resistant to a broad range of antibacterials [63]. *Stenotrophomonas* species have recently been reported in association with other coral species [64], [65], however their role in coral associated microbial communities has not been investigated.

Several phylotypes including unclassified *Bacteroidetes* and *Clostridria* were also identified in the ORFG study that were associated with apparently healthy *T. mesenterina* colonies and not detected at all in ASWS affected colonies, even in samples of the apparently healthy tissues. OTU 2921 is of particular interest because it was relatively abundant in one of the two apparently healthy coral colonies sampled. Given that its closest relative based on its 16S rRNA gene sequence was a *Clostridiaceae* species from fish gut and the fact that it was detected in only one of the two replicate healthy coral samples, it is possible that this phylotype originated from the gut of a commensal endolithic organism hidden inside the coral skeleton, or from a contamination of the coral sample with fish faeces. It is difficult to speculate about the role of the other *Bacteroidetes* and *Clostridria* identified in association with healthy corals in the health of the holobiont as none of these OTUs returned close matches to cultured bacteria in BLAST analyses.

### Conclusions

Australian Subtropical White Syndrome of *T. mesenterina* is a bacterial disease characterised by significant changes in the structure of the bacterial communities associated with the coral. These changes include shifts in the populations of specific organisms which may play important roles (either pathogenic or probiotic) in the health of the coral. Although there is now strong evidence indicating that ASWS has a bacterial aetiology, the identity of the bacterial pathogen or pathogens remains uncertain. Future work should focus on testing the ability of the *Vibrio* strains isolated in this study to infect *T. mesenterina* under controlled conditions, and also on enriching for the bacteria in the family *Rhodobactereaceae* that were identified in the culture-independent study as being significantly associated with disease lesions. It is important that these organisms are isolated in culture so that their role in the health of *T. mesenterina* and the development of ASWS can be investigated further.

## Supporting Information

Table S1 Relative abundance of all sequenced clones from the culture-independent study, including indicator species presented in Tables 2 and 3 and additional highly abundant clones.
(PDF)

Table S2 Accession numbers and details of BLAST and RDP classifier analysis for culturable bacteria isolated from *Turbinaria mesenterina.*
(PDF)
