# Supplementary material for: The Role of Coral-Associated Bacterial Communities in Australian Subtropical White Syndrome of Turbinaria mesenterina
Source: PLoS One. 2012 Sep 6;7(9):e44243. doi: 10.1371/journal.pone.0044243 (PMC3435329; doi:10.1371/journal.pone.0044243)
Supplement: Table S1 — Relative abundance of all sequenced clones from the culture-independent study, including indicator species presented in Tables 2 and 3 and additional highly abundant clones. (PDF) [file pone.0044243.s001.pdf]

Table S1. Relative abundance of all sequenced clones from the culture-independent study, including indicator species presented in tables 2 and 3 and additional highly abundant clones.

OTU	Acession number(s)	Nearest relative [accession number]	% Identity to nearest relative	RDP classifier taxon (80% confidence threshold)	Mean number of clones per sample category			
					H	M	D	C
269	EU780398<br>EU780397	Uncultured bacterium (Associated with *Lophelia pertusa* (cold water coral)) [AM911391]	91	Unclassified Gammaproteobacteria	3	2	0	7
1094	EU780418<br>EU780417<br>EU780402	*Muricauda* sp. (Marine isolate) [AY576776]	93	*Flavobacteriaceae* (Genus *Muricauda*)	1	0	0	11
2029	EU780404<br>EU780403	Uncultured marine bacterium [FJ594861]	99	*Flavobacteriaceae* (Unclassified)	0	<1	0	4.5
2802	EU780406<br>EU780405	Uncultured *Bactereroidetes* bacterium (marine bacterioplankton) [AY580710]	94	Unclassified *Bacteroidetes*	0	0	<1	5
2921	EU780400<br>EU780399	Uncultured bacterium (isolated from rainbow trout gut) [AM179918.1]	99	*Clostridiaceae (Genus Sarcina)*	0	1	0	54
2937	EU780408<br>EU780407	Uncultured bacterium (from pig gut) [AF371938.1]	95	Unclassified *Clostridales*	0	0	0	5
10	EU780286<br>EU780285	Escherichia coli DH1 [CP001637]	100	*Enterobacteriaceae* (genus *Escherichia*)	7.667	<1	0	1.5
165	EU780321<br>EU780320	Uncultured bacterium (nitrogen removing biofilm) [FM998994]	92	Unclassified bacteria	2	0	0	0
171	EU780302<br>EU780301	Uncultured alphaproteobacteria (*Monastrea annularis* (coral) tissue) [DQ200437]	93	Unclassified alphaproteobacteria	35.67	14	1.25	17
177	EU780290<br>EU780289	Sphingopyxis sp. MEBiC05060 (Marine sponge) [EU581700]	99	*Sphingomonadaceae* (genus *Sphingopyxis*)	4.333	0	0	<1
180	EU780316	Uncultured alphaproteobacteria (Body wall lesions of sea urchin) [AM930440]	99	Rhodobactereaceae (Unclassified)	1.667	1	<1	0
182	EU780318	Uncultured bacterium (marine microbial mat) [HQ153940]	97	Unclassified spirochaetales	2.333	0	0	0
184	EU780312<br>EU780311	Sphingopyxis sp. MEBiC05060 (Marine sponge) [EU581700]	98	*Sphingomonadaceae* (genus *Sphingopyxis*)	4.667	<1	<1	<1
187	EU780323<br>EU780322	Uncultured bacterium (Montastrea faveolata (coral)) [GU118637]	93	Unclassified Clostridia	2	0	0	0
191	EU780310<br>EU780309	Uncultured cyanobacterium (Antarctic lichen dominated cryptoendolithic community) [AY250874]	97	Cyanobacteria Family IV (genus GpIV)	5.667	<1	<1	0

* Sample categories - C - Completely healthy coral colonies with no sign of ASWS, H - Apparently healthy tissue of colonies affected by ASWS, M - Margin of ASWS disease lesions, D - Exposed coral skeleton adjacent to margin of disease lesion

Table S1. Relative abundance of all sequenced clones from the culture-independent study, including indicator species presented in tables 2 and 3 and additional highly abundant clones.

OTU	Acession number(s)	Nearest relative [accession number]	% Identity to nearest relative	RDP classifier taxon (80% confidence threshold)	Mean number of clones per sample category			
					H	M	D	C
195	EU780412<br>EU780411	Uncultured alpha proteobacteria (associated with whale carcass) [AY922246]	97	*Sphingomonadaceae* (Genus *Sphingopyxis*)	3	0	<1	<1
201	EU780300<br>EU780299	Uncultured bacterium (Montastrea franksi (coral)) [GU118721]	84	*Flavobacteriaceae* (unclassified)	59	3.5	<1	10.5
202	EU780288<br>EU780287	Delftia tsuruhatensis strain T7 (terephthalate-assimilating bacterium isolated from activated sludge) [NR_024786]	99	*Comamonadaceae* (genus *Delftia*)	5.667	<1	0	2
225	EU780306<br>EU780305	Uncultured bacteria (Monastrea franksi (coral)) [GU118721]	84	*Flavobacteriaceae* (unclassified)	15.67	0	0	2.5
231	EU780308<br>EU780307	Uncultured bacterium (sawmill sink water) [FJ716306]	97	*Chlorobiaceae* (genus *Prosthecochloris*)	7.667	0	0	0
242	EU780313	Uncultured bacterium (sawmill sink water) [FJ716306]	97	*Chlorobiaceae* (genus *Prosthecochloris*)	3.667	0	0	0
248	EU780410<br>EU780409	Uncultured Pseudomonas (soil isolate) [AJ295645.1|UPS295645]	86	Unclassified Gammaproteobacteria	4	<1	0	0
270	EU780414<br>EU780413	Escherichia coli 536 [CP000247]	99	*Enterobacteriaceae* Genus *Escherichia*	2	<1	<1	1
285	EU780416<br>EU780415	Uncultured *Bactereroidetes* bacterium (asscociated with sea urchin) [AY222319]	96	Unclassified *Bacteroidetes*	2	0	<1	1
320	EU780292<br>EU780291	Comamonas sp. M2T2B14 (mixture of bovine dung and urine, cow's milk, and yogurt undergoing aerobic fermentation) [GQ246691]	99	Comamonadaceae (genus Comamonas)	2.667	<1	0	1
348	EU780294<br>EU780293	Uncultured bacterium (wound of mouse skin) [HM820111]	99	Comamonadaceae (genus Comamonas)	2	0	0	<1
364	EU780324	Uncultured Ruegeria sp. (coral mucus) [FJ695550]	100	Rhodobactereaceae (genus Ruegeria)	1	<1	<1	0
389	EU780315<br>EU780314	Uncultured planctomycete (marine microbial mat) [AB294955]	99	Planctomycetaceae (genus Rhodopirellula)	1.333	1	0	1
390	EU780295	Uncultured bacterium [HM186874]	99	Xanthomonadaceae (genus Stenotrophomonas)	1.667	0	0	1

* Sample categories - C - Completely healthy coral colonies with no sign of ASWS, H - Apparently healthy tissue of colonies affected by ASWS, M - Margin of ASWS disease lesions, D - Exposed coral skeleton adjacent to margin of disease lesion

Table S1. Relative abundance of all sequenced clones from the culture-independent study, including indicator species presented in tables 2 and 3 and additional highly abundant clones.

**OTU**	**Acession number(s)**	**Nearest relative [accession number]**	**% Identity to nearest relative**	**RDP classifier taxon (80% confidence threshold)**	**Mean number of clones per sample category**			
					**H**	**M**	**D**	**C**
406	EU780319	Uncultured bacterium (Montastrea franksi (coral)) [GU118721]	84	Flavobacteriaceae (unclassified)	1.667	0	0	1
441	EU780296	Uncultured bacterium (Human intestinal pouch) [GQ157102]	99	Pseudomonadaceae (genus Pseudomonas)	1.667	0	0	1
843	EU780325	Uncultured bacterium (Porites asteroides (coral)) [GU118926]	92	Unclassified cyanobacteria	1.667	0	<1	0
989	EU780327 EU780326	Uncultured bacterium (marine sediment) [GQ259310]	96	Flavobacteriaceae (unclassified)	2	0	0	0
1014	EU780317	Uncultured alphaproteobacteria (Monastrea annularis (coral) tissue) [DQ200437]	94	Unclassified alphaproteobacteria	2.333	0	0	<1
1960	EU780298 EU780297	Uncultured proteobacteria (seawater) [Z31658]	92	Unclassified Gammaproteobacteria	2.333	0	0	0
236	EU780304 EU780303	Uncultured bacterium [HM186874]	99	*Xanthomonadaceae* (genus *Stenotrophomonas*)	20.33	4.5	0	21.5
302	EU780355	Uncultured bacterium (metallic artificial reef) [FJ594828]	96	Rhodobactereaceae (Unclassified)	<1	0	3	1
457	EU780357 EU780356	Shimia marina [AY962292]	99	Rhodobactereaceae (Genus Shimia)	0	<1	3.5	0
468	EU780344 EU780343	Uncultured bacterium (leaf packs in woodland stream) [GU568990]	94	Chloroplast of Bacillariophyta (diatom)	0	1	5.25	0
487	EU780331	Uncultured bacterium (black band diseased coral) [EF123421]	98	Rhodobactereaceae (Unclassified)	<1	1	3	2
503	EU780371 EU780370	Uncultured bacterium (metallic artificial reef)	99	Cyanobacteria Family IV (genus GpIV)	<1	<1	1.25	0
524	EU780381 EU780382	Amphitrea atlantica [AM156910]	97	Oceanospirillaceae (Genus Amphritea)	0	<1	1.25	0
536	EU780336	Uncultured bacterium (leaf packs in woodland stream) [GU568990]	95	Chloroplast of *Bacillariophyta* (diatom)	0	<1	1.25	0
546	EU780362	Uncultured alphaproteobacterium (marine sediment) [DQ289901]	96	Rhodobactereaceae (Unclassified)	0	1	2.25	0

* Sample categories - C - Completely healthy coral colonies with no sign of ASWS, H - Apparently healthy tissue of colonies affected by ASWS, M - Margin of ASWS disease lesions, D - Exposed coral skeleton adjacent to margin of disease lesion

Table S1. Relative abundance of all sequenced clones from the culture-independent study, including indicator species presented in tables 2 and 3 and additional highly abundant clones.

OTU	Acession number(s)	Nearest relative [accession number]	% Identity to nearest relative	RDP classifier taxon (80% confidence threshold)	Mean number of clones per sample category			
					H	M	D	C
560	EU780383 EU780384	Uncultured bacterium (diseased tissue of Montastrea faveolata (coral)) [FJ203036]	99	Unclassified Gammaproteobacteria	0	<1	1.25	0
566	EU780372	Uncultured bacterium (leaf packs in woodland stream) [GU568990]	95	Chloroplast of *Bacillariophyta* (diatom)	0	1	1.5	0
604	EU780385 EU780386 EU780387	Uncultured organism associated with coral Diploria strigosa (coral) [GU119622]	98	Chloroplast of *Bacillariophyta* (diatom)	0	<1	1.25	0
685	EU780354 EU780353	Uncultured diatom chloroplast (assoiciated with Montastrea faveolata (coral)) [FJ403099]	94	Chloroplast of Bacillariophyta (diatom)	0	0	4	0
706	EU780373	Uncultured cyanobacterium (intertidal thrombolite) [GQ483866]	98	Cyanobacteria Family IV (genus GpIV)	0	0	2	0
716	EU780338 EU780337	Uncultured bacterium (arctic stream) [FJ849120]	98	Chloroplast of *Bacillariophyta* (diatom)	<1	<1	9.5	0
724	EU780346 EU780345	Uncultured bacterium (leaf packs in woodland stream) [GU568990]	95	Chloroplast of *Bacillariophyta* (diatom)	0	<1	5.5	0
734	EU780374	Uncultured bacterium (diseased tissue of Montastrea faveolata (coral)) [FJ203308]	99	Chloroplast of *Bacillariophyta* (diatom)	0	0	2	0
753	EU780364	Chloroplast of uncultured eukaryote [EF491335]	99	Chloroplast of *Bacillariophyta* (diatom)	0	0	2.5	0
759	EU780335 EU780334	Uncultured diatom chloroplast (sediment) [FM242284]	99	Chloroplast of *Bacillariophyta* (diatom)	0	0	2.5	0
763	EU780333 EU780332	Uncultured eukaryote plastid [HM262878]	97	Chloroplast of *Bacillariophyta* (diatom)	0	0	4.75	0
806	EU780329 EU780328	Uncultured bacterium (marine microbial mat) [GQ441254]	99	Chloroplast of *Bacillariophyta* (diatom)	0	<1	4.75	0
845	EU780342 EU780341	Uncultured bacterium (marine biofilm) [GU066447]	96	Flavobacteriaceae (genus Winogradskyella)	<1	0	6.25	0
846	EU780350 EU780349	Uncultured bacterium (leaf packs in woodland stream) [GU568990]	94	Chloroplast of *Bacillariophyta* (diatom)	0	<1	5	0
862	EU780390 EU780330	Rhodobactereaceae bacterium [AY177714]	98	*Rhodobactereaceae* (Genus *Roseovarius*)	0	0	1.5	0

* Sample categories - C - Completely healthy coral colonies with no sign of ASWS, H - Apparently healthy tissue of colonies affected by ASWS, M - Margin of ASWS disease lesions, D - Exposed coral skeleton adjacent to margin of disease lesion

Table S1. Relative abundance of all sequenced clones from the culture-independent study, including indicator species presented in tables 2 and 3 and additional highly abundant clones.

OTU	Acession number(s)	Nearest relative [accession number]	% Identity to nearest relative	RDP classifier taxon (80% confidence threshold)	Mean number of clones per sample category			
					H	M	D	C
872	EU780389 EU780388	Vibrio communis [GU078673.1]	99	*Vibrionaceae* (Genus *Vibrio*)	0	<1	1	<1
874	EU780352 EU780351	Uncultured bacterium (coastal microbial mat) [GQ441312]	97	Chloroplast of *Bacillariophyta* (diatom)	0	0	5	0
1015	EU780340 EU780339	Thalassobius sp. SC027 [FJ889533]	97	Rhodobactereaceae (Unclassified)	<1	1	5.75	0
1206	EU780369	Uncultured bacterium (marine hydrocarbon seep) [GU584528]	97	Rhodobactereaceae (Unclassified)	<1	0	1.75	<1
1264	EU780361 EU780360	Uncultured bacterium (marine hydrocarbon seep) [GU584528]	97	Rhodobactereaceae (Unclassified)	<1	1	2.5	0
1427	EU780366 EU780365	Uncultured deep-sea bacterium [AM997664]	95	Unclassified Flavobacteriales	0	0	2.5	0
1489	EU780367	Uncultured baterium (diseased tissue of Montastrea faveolata (coral)) [FJ203253]	99	Rhodobactereaceae (Unclassified)	<1	1.5	1.5	0
1575	EU780375	Uncultured alphaproteobacterium (submerged artificial inert substrate) [EU005320]	96	Rhodobactereaceae (Unclassified)	0	<1	1.75	0
1740	EU780358	Uncultured baterium (diseased tissue of Montastrea faveolata) [FJ203103]	99	Rhodobactereaceae (Unclassified)	<1	<1	3	0
2073	EU780348 EU780347	Uncultured bacterium (Montastrea faveolata (coral) in aqaurium) [FJ202903]	99	Clostridiales Incertae Sedis XII (genus Fusibacter)	0	1	5	0
2112	EU780391	Uncultured bacterium associated with diseased tissue of Montastrea faveolata (coral) [FJ203253.1]	99	*Rhodobactereaceae* (Unclassified)	0	<1	1.25	0
2365	EU780392	Uncultured bacterium associated with diseased tissue of Montastrea faveolata (coral) [FJ203196]	99	*Cohaesibacteraceae* (Genus *Cohaesibacter*)	<1	0	1.25	0
2469	EU780393	Uncultured bacterium associated with diseased tissue of Montastrea faveolata(coral) [FJ203089.1]	99	*Rhodobactereaceae* (Unclassified)	0	0	1.5	0
2476	EU780395 EU780394	Uncultured gammaproteobacterium associated with body wall lesions of sea urchin [AM930470]	99	*Oceanospirillaceae* (Genus *Neptunomonas*)	0	0	1.5	0
2486	EU780359	Shimia marina [AY962292]	99	Rhodobactereaceae (Genus Shimia)	0	0	3.25	0
2499	EU780363	Uncultured bacterium (marine hydrocarbon seep) [GU584528]	97	Rhodobactereaceae (Unclassified)	0	0	2.75	0

* Sample categories - C - Completely healthy coral colonies with no sign of ASWS, H - Apparently healthy tissue of colonies affected by ASWS, M - Margin of ASWS disease lesions, D - Exposed coral skeleton adjacent to margin of disease lesion

Table S1. Relative abundance of all sequenced clones from the culture-independent study, including indicator species presented in tables 2 and 3 and additional highly abundant clones.

OTU	Acession number(s)	Nearest relative [accession number]	% Identity to nearest relative	RDP classifier taxon (80% confidence threshold)	Mean number of clones per sample category			
					**H**	**M**	**D**	**C**
2509	EU780368	Uncultured bacteroidetes bacterium (body wall lesions of sea urchin) [AM930459]	95	Cryomorphaceae (unclassified)	0	0	2.5	0
2522	EU780376 EU780377	Uncultured bacterium (diseased tissue of Montastrea faveolata (coral)) [FJ203089]	99	Rhodobactereaceae (Unclassified)	0	0	2	0
2527	EU780396	Uncultured bacterium (from marine hydrocarbon seep) [GU584528.1]	96	*Rhodobactereaceae* (Unclassified)	0	0	1.5	0
467	EU780379 EU780380	Uncultured bacterium from coral mucus [FJ930674]	96	Unclassified Gammaproteobacteria	0	1	1	0
518	EU780378	Uncultured alphaproteobacterium (marine sediment) [DQ289933]	98	Rhodobactereaceae (Unclassified)	0	1	1.25	0
175	EU780226 EU379566	Uncultured alpha proteobacterium from squid mucus [AJ633968]	99	*Rhodobactereaceae* (Genus *Silicibacter*)	56	129	34	21
204	EU780422 EU780421	Marine bacterium ATAM173a_16 [AF359531] isolated from a culture of Alexandrium tamarense (dinoflagellate)	96	*Rhodobactereaceae* (unclassified)	2.333	7	4	5.5
210	EU780230 EU780229	*Thalassobius mediterraneus* [AJ878874]	99	*Rhodobactereaceae* (unclassified)	3	12	5	1
265	EU780267 EU780268	Uncultured bacterium (from sponge) [AY845242]	98	*Rhodobactereaceae* (Genus *Roseobacter*)	10	26	19	4
307	EU780276 EU780275	Rhodobacteraceae bacterium [AY177714]	*98*	*Rhodobactereaceae* (Genus *Roseovarius*)	1.667	2.5	1	1
329	EU780228 EU780227	Rhodobacteraceae bacterium [AY177714]	98	*Rhodobactereaceae* (Genus *Roseovarius*)	1.667	17	3.5	2.5
384	EU780250 EU780249	Rhodobacteraceae bacterium [AY177714]	98	*Rhodobactereaceae* (Genus *Roseovarius*)	1	2	<1	0
388	EU780274 EU780273	Rhodobacteraceae bacterium [AJ810844]	*96*	*Rhodobactereaceae* (unclassified)	<1	4	1.75	0
429	EU780238 EU780237	Algal Chloroplast [X76084]	92	Unclassified cyanobacteria	1.333	8	<1	<1
449	EU780234 EU780233	Uncultured alpha proteobacterium (from squid) [AJ633989]	96	*Rhodobactereaceae* (unclassified)	0	7	2.5	0
452	EU780244 EU780243	Uncultured bacterium (from seafloor sediment) [AY35417]	90	Unclassified Gammaproteobacteria	0	5	<1	0

* Sample categories - C - Completely healthy coral colonies with no sign of ASWS, H - Apparently healthy tissue of colonies affected by ASWS, M - Margin of ASWS disease lesions, D - Exposed coral skeleton adjacent to margin of disease lesion

Table S1. Relative abundance of all sequenced clones from the culture-independent study, including indicator species presented in tables 2 and 3 and additional highly abundant clones.

OTU	Acession number(s)	Nearest relative [accession number]	% Identity to nearest relative	RDP classifier taxon (80% confidence threshold)	Mean number of clones per sample category			
					H	M	D	C
459	EU780246 EU780245	Uncultured epsilon proteobacterium (deep sea isolate) [AB113178]	90	Unclassified Bacteria	0	4.5	<1	0
469	EU780272 EU780271	Marine alpha proteobacterium [AJ391182]	93	*Rhodobactereaceae* (unclassified)	2	5	2.25	1.5
480	EU780232 EU780231	*Thalassobius mediterraneus* [AJ878874]	99	*Rhodobactereaceae* (unclassified)	<1	10	3.5	2
491	EU780270 EU780269	Uncultured bacterium (sea sediment) [AY373402]	93	Unclassified Gammaproteobacteria	<1	10	5.5	0
497		Rhodobacteraceae bacterium (from fish farm biofilm) [AY962292]	96	*Rhodobactereaceae* (unclassified)	<1	6.5	3.75	3
521	EU780240 EU780239	Uncultured bacterium (from sponge) [AY845242]	98	*Rhodobactereaceae* (Genus *Roseovarius*)	1.333	5	2	0
525	EU780258 EU780257	Uncultured *Thiothrix* (from marine amphipod) [AY426613]	92	*Thiotrichaceae* (Genus *Thiotrix*)	0	2	<1	0
530	EU780236 EU780235	*Roseovarius crassostereae* [AF114484] tied with CVSP bacterium [AF114485] (both associated with juvenile oyster disease)	97	*Rhodobactereaceae* (Genus *Jannaschia)*	<1	11	2	0
534	EU780260 EU780259	Uncultured alpha proteobacterium (associated with black band disease in corals) [AF473915]	97	*Rhodobactereaceae* (unclassified)	0	3	<1	0
539	EU780254 EU780253	Uncultured Bacteroidetes (from hypersaline microbial mat) [DQ330308]	89	*Saprospiraceae* (Unclassified)	0	3.5	0	0
562	EU780278 EU780277	Flavobacterium (artic marine sediment) [DQ514308]	93	*Flavobacteriaceae (Genus Algibacter)*	0	3.5	1.25	1
601	EU780420 EU780419	Uncultured gamma proteobacteria (associated with coral tissue) [DQ200616]	89	Unclassified Gammaproteobacteria	0	2	2	0
609	EU780262 EU780261	Marine Flavobacterium [AY028207]	95	*Flavobacteriaceae (Unclassified)*	0	2	<1	0
616	EU780248 EU780247	*Thiothrix* sp. (marine isolate) [DQ067608]	96	*Thiotrichaceae* (Genus *Thiotrix*)	0	5.5	<1	0
851	EU780280 EU780279	*Plectonema* sp. (cyanobacterium) [AF091110]	94	Unclassified cyanobacteria	0	3.5	1.5	0

* Sample categories - C - Completely healthy coral colonies with no sign of ASWS, H - Apparently healthy tissue of colonies affected by ASWS, M - Margin of ASWS disease lesions, D - Exposed coral skeleton adjacent to margin of disease lesion

Table S1. Relative abundance of all sequenced clones from the culture-independent study, including indicator species presented in tables 2 and 3 and additional highly abundant clones.

OTU	Acession number(s)	Nearest relative [accession number]	% Identity to nearest relative	RDP classifier taxon (80% confidence threshold)	Mean number of clones per sample category			
					H	M	D	C
1017	EU780282<br>EU780281	Uncultured marine bacterium [DQ071069]	96	*Verrucomicrobiaceae* (Genus *Verrucomicrobium*)	1	1.5	<1	0.5
1310	EU780252<br>EU780251	Three-way tie; Pleurocapsa sp. [X78681], Myxosarcina sp. [AJ344561], Pleurocapsa sp. [AJ344561	95	Unclassified subsection 2 Cyanobacteria	<1	3	<1	0
1574	EU780256<br>EU780255	*Thalassobius mediterraneus* [AJ878874]	97	*Rhodobactereaceae* (unclassified)	0	2.5	<1	<1
2011	EU780264<br>EU780263	Marine bacterium Flavobacterium [AY028207]	95	*Flavobacteriaceae* (Unclassified)	0	2.5	<1	0
2049	EU780284<br>EU780283	*Roseovarius crassostereae* [AF114484] tied with CVSP bacterium [AF114485] (both associated with juvenile oyster disease)	96	*Rhodobactereaceae* (unclassified)	0	2	1	0
2051	EU780242<br>EU780241	Marine bacterium Flavobacterium [AY028207]	95	*Flavobacteriaceae* (Unclassified)	0	9.5	<1	0
2094	EU780266<br>EU780265	*Thiothrix* sp. [DQ067608]	96	*Thiotrichaceae* (Genus *Thiotrix*)	0	3	0	0

* Sample categories - C - Completely healthy coral colonies with no sign of ASWS, H - Apparently healthy tissue of colonies affected by ASWS, M - Margin of ASWS disease lesions, D - Exposed coral skeleton adjacent to margin of disease lesion
