# Supplementary material for: The Role of Coral-Associated Bacterial Communities in Australian Subtropical White Syndrome of Turbinaria mesenterina
Source: PLoS One. 2012 Sep 6;7(9):e44243. doi: 10.1371/journal.pone.0044243 (PMC3435329; doi:10.1371/journal.pone.0044243)
Supplement: Table S2 — Accession numbers and details of BLAST and RDP classifier analysis for culturable bacteria isolated from Turbinaria mesenterina. (PDF) [file pone.0044243.s002.pdf]

Table S2. Accession numbers and details of BLAST and RDP classifier analysis for culturable bacteria isolated from *Turbinaria mesenterina*

Isolate Name	Accession number(s)	Sample category*	RDP Classifier Taxon (80% confidence threshold)	Nearest Relative [accession number]	% Identity to nearest relative
**2.3.053CC3**	EU267644	C	Flavobacteriaceae (genus *Winogradskyella*)	Winogradskyella poriferorum strain UST030701-295 [AY848823]	97%
**2.3.053CC4**	EU267623	C	Rhodobacteraceae (genus *Ruegeria*)	*Ruegeria atlantica* [JN128252]	100%
**4.5.0412CS1**	EU276994	C	Vibrionaceae (genus *Vibrio*)	Bacterium WP2ISO14 [DQ985841]	97%
**4.5.0412CS2a**	EU267654	C	Vibrionaceae (genus *Vibrio*)	Bacterium WP2ISO6 [DQ985833]	98%
**4.5.0412CS3**	EU267612	C	Vibrionaceae (genus *Vibrio*)	Bacterium WP3ISO9 [DQ985870]	99%
**4.5.0412CS4**	EU267616	C	Vibrionaceae (genus *Vibrio*)	Bacterium WP2ISO14 [DQ985841]	99%
**4.5.0412CS7**	EU276996	C	Vibrionaceae (genus *Photobacterium*)	Photobacterium lutimaris strain DF-42 [DQ534014]	97%
**4.5.0412CS8**		C	Vibrionaceae (genus *Vibrio*)	Vibrio sp. V639 [DQ146989]	98%
**4.5.0412CS9**	EU267624	C	Vibrionaceae (genus *Photobacterium*)	Photobacterium sp. YL2 [EF187015]	99%
**4.5.0412CS11**	EU277000	C	Vibrionaceae (genus *Vibrio*)	Vibrio sp. V051 [DQ146974]	
**4.5.0412CS12**	EU276992	C	Rhodobacteraceae (genus *Paracoccus*)	Paracoccus sp. JL1105 [DQ985064]	97%
**2.3.052HS1**	EU267665	H	Unclassified Gammaproteobacteria	Uncultured Vibrio sp. clone 12 [AY785252]	93%
**2.3.052HS2**	EU267640	H	Vibrionaceae (genus *Vibrio*)	Vibrio sp. YASM14 [DQ314529]	97%
**2.3.053HC2**	EU267668	H	Moraxellaceae (genus *Psychrobacter*)	Psychrobacter celer strain KOPRI24932 [EF101550]	99%
**2.3.053HS1**	EU267669	H	Unclassified Rhodobacteraceae	Rhodobacteraceae bacterium CL-TA03 [AY962292]	97%
**2.3.053HS2**	EU267629	H	Shewanellaceae (genus *Shewanella*)	Shewanella sp. C321 [DQ005902]	99%
**2.3.053HS3**	EU267634	H	Vibrionaceae (genus *Vibrio*)	Vibrio sp. HB-8 [AY876051]	97%
**2.3.053HS5**	EU267635	H	Pseudoalteromonadaceae (genus *Pseudoalteromonas*)	Marine bacterium LMG1 [AY082666]	98%

* Sample categories - C - Completely healthy coral colonies with no sign of ASWS, H - Apparently healthy tissue of colonies affected by ASWS, M - Margin of ASWS disease lesions, D - Exposed coral skeleton adjacent to margin of disease lesion

Table S2. Accession numbers and details of BLAST and RDP classifier analysis for culturable bacteria isolated from *Turbinaria mesenterina*

Isolate Name	Accession number(s)	Sample category*	RDP Classifier Taxon (80% confidence threshold)	Nearest Relative [accession number]	% Identity to nearest relative
**4.5.0412HS1**	EU267656	H	Vibrionaceae (genus *Vibrio*)	Bacterium WP2ISO14 [DQ985841]	99%
**4.5.0412HS3**	EU277002	H	Vibrionaceae (genus *Vibrio*)	Vibrio sp. A2 [EF467288	98%
**4.5.0412HS4**	EU276998	H	Vibrionaceae (genus *Vibrio*)	Bacterium WP2ISO14 [DQ985841]	97%
**2.3.052MS2**	EU267650	M	unclassified Gammaproteobacteria	Uncultured Vibrio sp. clone 12 [AY785252]	94%
**2.3.052MS3**	EU267662	M	Vibrionaceae (genus *Vibrio*)	Vibrio harveyi strain LB4 [DQ146935]	99%
**2.3.052MS5**	EU267643	M	Vibrionaceae (genus *Vibrio*)	Bacterium WP2ISO12 [DQ985839]	99%
**2.3.053MS1**	EU267636	M	Unclassified Rhodobacteraceae	Uncultured alpha proteobacterium clone JL-ECS-X8 [AY663968]	96%
**2.3.053MS2**	EU267638	M	Rhodobacteraceae (genus *Silicibacter*)	Rhodobacteraceae bacterium CL-TA03 [AY962292]	94%
**2.3.053MS3**	EU267639	M	Vibrionaceae (genus *Vibrio*)	Vibrio harveyi strain LB4 [DQ146935]	98%
**2.3.053MS4**	EU267647	M	Vibrionaceae (genus *Vibrio*)	Vibrio harveyi strain LB4 [DQ146935]	99%
**2.3.053MS5**	EU267670	M	Vibrionaceae (genus *Vibrio*)	Bacterium WP2ISO6 [DQ985833]	98%
**4.5.0412MS1**	EU276989	M	Vibrionaceae (genus *Vibrio*)	Bacterium WP3ISO9 [DQ985870]	98%
**4.5.0412MS2**	EU276993	M	Vibrionaceae (genus *Vibrio*)	Vibrio sp. PH1 [AF513461]	97%
**4.5.0412MS3**	EU276999	M	Vibrionaceae (genus *Vibrio*)	Vibrio harveyi strain LB4 [DQ146935]	97%
**4.5.0412MS4**	EU267657 EU276995	M	Vibrionaceae (genus *Vibrio*)	Vibrio sp. BA2 [EF187016]	97%
**4.5.0412MS5**	EU276990	M	Vibrionaceae (genus *Vibrio*)	Bacterium WP3ISO9 [DQ985870]	98%
**4.5.0412MS6**	EU267653 EU259885	M	Vibrionaceae (genus *Vibrio*)	Bacterium WP3ISO10 [DQ985871]	99%
**4.5.0412MS7**	EU267659	M	Vibrionaceae (genus *Vibrio*)	Bacterium WP2ISO9 [DQ985836]	98%

* Sample categories - C - Completely healthy coral colonies with no sign of ASWS, H - Apparently healthy tissue of colonies affected by ASWS, M - Margin of ASWS disease lesions, D - Exposed coral skeleton adjacent to margin of disease lesion

Table S2. Accession numbers and details of BLAST and RDP classifier analysis for culturable bacteria isolated from *Turbinaria mesenterina*

Isolate Name	Accession number(s)	Sample category*	RDP Classifier Taxon (80% confidence threshold)	Nearest Relative [accession number]	% Identity to nearest relative
**2.3.052DS1**	EU267664	D	Ferrimonidaceae (genus *Ferrimonas*)	Ferrimonas kyonanensis [AB245514]	97%
**2.3.052DS2**	EU267642	D	Vibrionaceae (genus *Vibrio*)	Vibrio sp. R-14939, strain R-14939 [AJ316187]	96%
**2.3.052DS3**	EU267648	D	Vibrionaceae (genus *Vibrio*)	Vibrio sp. V639 [DQ146989]	98%
**2.3.052DS4**	EU267630	D	Vibrionaceae (genus *Vibrio*)	Vibrio harveyi strain LB4 [DQ146935]	99%
**2.3.052DS5**	EU267649	D	Unclassified Rhodobacteraceae	Rhodobacteraceae bacterium CL-TA03 [AY962292]	96%
**2.3.053DC1**	EU267631	D	Vibrionaceae (genus *Vibrio*)	Vibrio harveyi strain LB4 [DQ146935]	97%
**2.3.053DC2**	EU267632	D	Rhodobacteraceae (genus *Silicibacter*)	Rhodobacteraceae bacterium CL-TA03 [AY962292]	96%
**2.3.053DC3**	EU267666	D	Vibrionaceae (genus *Vibrio*)	Uncultured gamma proteobacterium clone UA07 [DQ269050]	97%
**2.3.053DC4**	EU267651	D	Unclassified Rhodobacteraceae	Rhodobacteraceae bacterium CL-TA03 [AY962292]	98%
**2.3.053DC5**		D	Unclassified Gammaproteobacteria	Vibrio sp. V068 [DQ146975]	97%
**2.3.053DC6**	EU267633	D	Vibrionaceae (genus *Vibrio*)	Bacterium WP2ISO12 [DQ985839]	94%
**2.3.053DC8**	EU267637	D	Unclassified Rhodobacteraceae	Unidentified bacterium clone WP2OTU8 [DQ985908]	96%
**2.3.053DC9**	EU267645	D	Vibrionaceae (genus *Vibrio*)	Vibrio sp. HB-8 [AY876051]	98%
**2.3.053DS1**	EU267646	D	Unclassified Rhodobacteraceae	Rhodobacteraceae bacterium CL-TA03 [AY962292]	98%
**2.3.053DS2**	EU267641	D	Pseudoalteromonadaceae (genus *Pseudoalteromonas*)	Unidentified bacterium clone WP2OTU33 [DQ985917]	96%

* Sample categories - C - Completely healthy coral colonies with no sign of ASWS, H - Apparently healthy tissue of colonies affected by ASWS, M - Margin of ASWS disease lesions, D - Exposed coral skeleton adjacent to margin of disease lesion

Table S2. Accession numbers and details of BLAST and RDP classifier analysis for culturable bacteria isolated from *Turbinaria mesenterina*

Isolate Name	Accession number(s)	Sample category*	RDP Classifier Taxon (80% confidence threshold)	Nearest Relative [accession number]	% Identity to nearest relative
**2.3.053DS3**	EU267667	D	Vibrionaceae (genus *Vibrio*)	Vibrio sp. LC1-199 [AB239472]	99%
**2.3.053DS4**	EU267652	D	Incertae sedis 7 (genus *Agarivorans*)	Agarivorans albus, strain:MKT112 [AB076562]	96%
**4.5.0412DS1**	EU267608	D	Vibrionaceae (genus *Vibrio*)	Bacterium WP2ISO14 [DQ985841]	97%
**4.5.0412DS2**	EU267617	D	Vibrionaceae (genus *Vibrio*)	Bacterium WP3ISO9 [DQ985870]	97%
**4.5.0412DS3a**	EU276997	D	Vibrionaceae (genus *Vibrio*)	Uncultured bacterium isolate LCPISO4 [DQ831087]	95%
**4.5.0412DS4**	EU267655	D	Vibrionaceae (genus *Vibrio*)	Bacterium WP2ISO14 [DQ985841]	98%
**4.5.0412DS6**	EU276991	D	Vibrionaceae (genus *Vibrio*)	Bacterium WP3ISO9 [DQ985870]	96%
**4.5.0412DS8**	EU267609	D	Pseudoalteromonadaceae (genus *Pseudoalteromonas*)	Bacterium WP3ISO11 [DQ985872]	96%
**4.5.0412DS11**	EU277001	D	Vibrionaceae (genus *Enterovibrio*)	Enterovibrio norvegicus strain LMG 19842 [AJ437193]	98%

* Sample categories - C - Completely healthy coral colonies with no sign of ASWS, H - Apparently healthy tissue of colonies affected by ASWS, M - Margin of ASWS disease lesions, D - Exposed coral skeleton adjacent to margin of disease lesion
